# Estimation of persistent inward currents contribution to inspiratory motoneuron firing in humans

**DOI:** 10.1113/JP290663

**Published:** 2026-07-22

**Authors:** Ricardo N. O. Mesquita, Simon C. Gandevia, Janet L. Taylor, Anna L. Hudson, Jane E. Butler

**Affiliations:** ^1^ Department of Laboratory Medicine, Division of Clinical Physiology Karolinska Institutet Stockholm Sweden; ^2^ Unit of Clinical Physiology Karolinska University Hospital Stockholm Sweden; ^3^ School of Medical and Health Sciences Edith Cowan University Perth Australia; ^4^ Kolling Institute Sydney NSW Australia; ^5^ Faculty of Medicine and Health, School of Medical Sciences The University of Sydney Sydney NSW Australia; ^6^ Centre for Precision Health, School of Medical and Health Sciences Edith Cowan University Perth WA Australia; ^7^ Flinders Health and Medical Research Institute Flinders University Adelaide SA Australia

**Keywords:** bistability, input–output function, motoneurone, motor neuron, noradrenaline, phrenic, serotonin

## Abstract

**Abstract:**

Persistent inward currents (PICs) are known to prolong firing of limb‐muscle motoneurons. Although there is immunohistochemical evidence of PIC channels in respiratory motoneurons, it is unknown whether PICs contribute to their firing prolongation. Intramuscular electromyographic signals were recorded from human inspiratory muscles to identify motor unit (MU) activity. Diaphragm MUs were identified during quiet breathing (*n* = 7; one female) and MUs from the 1st, 3rd, and 5th parasternal intercostal muscles during quiet (no lung volume feedback) and voluntary (triangular‐shaped lung volume feedback) breathing (*n* = 5 males). PIC contribution to firing prolongation was estimated via quantification of firing hysteresis (paired MU analysis; ∆*F*) and firing symmetry in relation to peak volume (duration ratio). Diaphragm was the only muscle in which MUs exhibited ∆*F* scores significantly greater than 1 Hz, suggesting a possible PIC contribution. The proportion of MUs firing into expiration (duration ratio < 1) was higher in diaphragm than in the 1st and 3rd intercostal muscles, but not different to the 5th. Duration ratios were higher in voluntary compared to quiet breaths in the 3rd and 5th intercostals, suggesting less firing prolongation. However, ∆*F* was not different between quiet and voluntary breaths in parasternal intercostal MUs. These findings suggest that PICs contribute to firing prolongation in diaphragm MUs during quiet breathing. However, PIC‐like behaviours were not evident in parasternal intercostal MUs firing during either quiet or voluntary breathing. These data underscore potentially different levels of neuromodulation across inspiratory muscles and further advance our understanding of the neural mechanisms regulating respiratory muscle control.

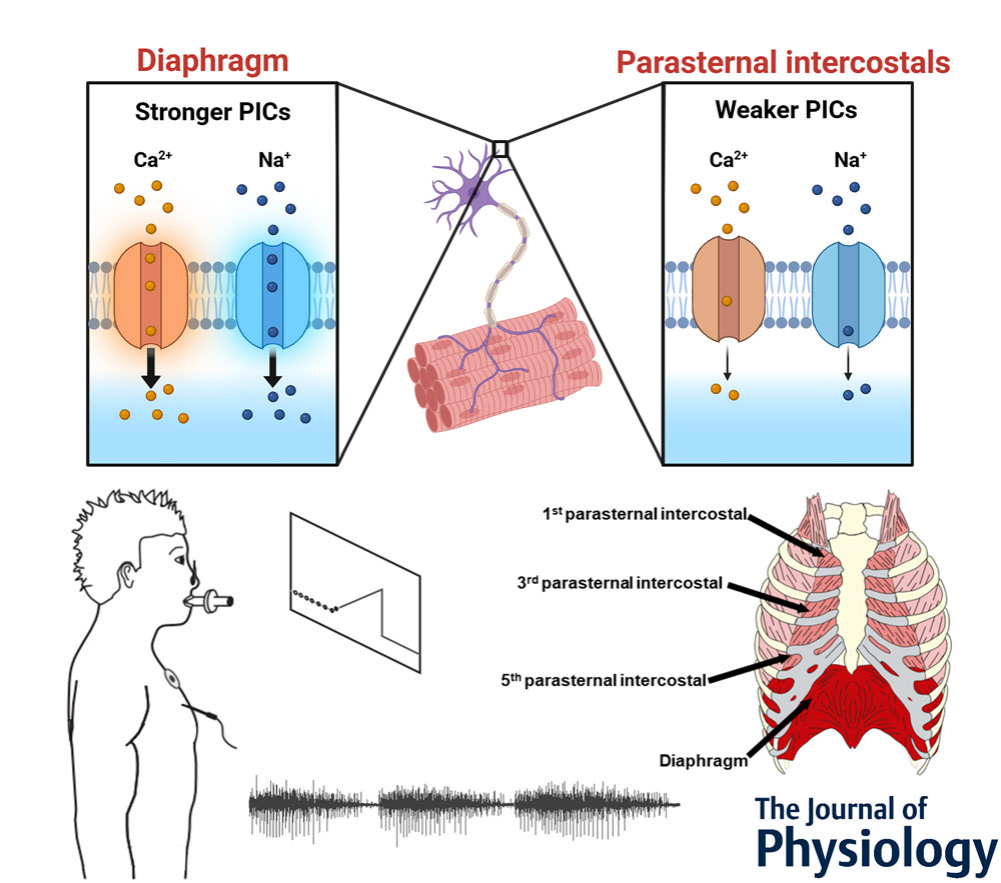

**Key points:**

- Spinal motoneurons transmit signals to muscles to regulate their contraction, and the intrinsic excitability of motoneurons is enhanced by persistent inward currents (PICs). PICs consist of a persistent flow of sodium and calcium ions into the motoneuron, which can help initiate, accelerate and prolong its firing.
- We investigated PIC contributions to motoneuron firing in human inspiratory muscles by analysis of diaphragm and parasternal intercostal motor unit activity during involuntary (quiet) and voluntary breathing.
- Diaphragm motor units showed a magnitude of firing prolongation consistent with a significant role of PICs in this muscle, whereas no such effect was observed in parasternal intercostal muscles.
- These findings suggest a differential contribution of PICs to motoneuron firing across different inspiratory muscles.
- Our study enhances understanding of respiratory muscle control and motivates future efforts to validate, further estimate or modulate possible PIC contributions to respiratory motoneuron firing in humans and animal models.

## Graphical abstract

**Abstract figure legend** Nerve cells in the spinal cord (alpha motoneurons) send signals to breathing muscles to control their contraction. The strength of these signals is boosted by persistent inward currents (PICs), which consist of a sustained flow of calcium (Ca^2+^) and sodium (Na^+^) ions into the motoneuron that helps start, amplify and sustain motoneuron activity. We recorded the activity of individual motor units in inspiratory muscles during both quiet and voluntary breathing in humans. Diaphragm motor units showed firing patterns consistent with PIC contribution, whereas parasternal intercostal motor units did not, suggesting that PICs play a greater role in driving the activity of diaphragm motoneurons than the parasternal intercostals. These findings suggest that PICs do not contribute equally to the control of all breathing muscles, revealing unexpected differences in how the nervous system controls each breathing muscle.

## Introduction

From the first fetal breathing movements to the final gasp, respiratory motoneurons are activated rhythmically to induce contractions of the skeletal muscles for ventilation. The firing behaviour of respiratory motoneurons is shaped by the organisation of ionotropic synaptic inputs from descending and reflex pathways (Butler, 2007; Butler et al., 2025). However, the extent to which neuromodulatory mechanisms influence the intrinsic excitability of respiratory motoneurons and introduce non‐linearity into their synaptic input–output relationship remains unclear. In limb motoneurons, such non‐linearities are primarily driven by persistent inward currents (PICs), which are generated by fast‐activating voltage‐gated sodium and slow‐activating calcium channels (Binder et al., 2020; Heckman et al., 2005, 2009; Mesquita et al., 2024). PICs are crucial for initiating, accelerating and sustaining motoneuron firing, and their activity varies with levels of the monoamines serotonin (Hounsgaard et al., 1988) and noradrenaline (Conway et al., 1988) released onto the motoneurons. PICs cannot be directly measured in humans but their contribution to motoneuron firing has been estimated and modulated in several limb‐innervating motoneuron pools (Mesquita et al., 2024). However, the potential contribution of PICs to human respiratory motoneuron firing remains unexplored.

Although there is immunohistochemical evidence for the presence of persistent calcium channels in feline phrenic motoneurons (Enríquez Denton et al., 2012; Zhang et al., 2008), PICs appear to contribute minimally to the voltage‐dependent amplification of synaptic inputs (i.e. the increase in peak depolarisation amplitude during inspiration at more depolarised membrane potentials), with NMDA receptor‐mediated synaptic currents playing a more prominent role (Enríquez Denton et al., 2012). Amplification of synaptic inputs has commonly been associated with an acceleration of firing upon motoneuron recruitment (Binder et al., 2020). Yet, this is just one possible aspect of the multifaceted effects of PICs and other intrinsic properties on human motoneuron output (Binder et al., 2020; Chardon et al., 2023; Mesquita et al., 2024). Other non‐linear firing patterns have been widely described in human motor unit recordings: firing hysteresis (Gorassini et al., 2002a), firing saturation (Fuglevand et al., 2015), warm‐up (Gorassini et al., 2002b) and self‐sustained firing (Gorassini et al., 1998). Interestingly, plateau potentials were observed in the study by Enríquez Denton et al. (2012) in expiratory motoneurons in the thoracic region presumably innervating expiratory intercostal or abdominal muscles. These plateau potentials are prolonged depolarisations that persist after the end of synaptic input and can lead to self‐sustained firing (Lee & Heckman, 1998). Notably, they were abolished under barbiturate anaesthesia, a known suppressor of PICs (Guertin & Hounsgaard, 1999), supporting their role in the generation of this intrinsic excitability. Although animal data suggest that PICs contribute minimally to the amplification of synaptic inputs in diaphragmatic motoneuron firing, the diversity of non‐linear firing patterns that PICs can produce, along with their potential presence in other respiratory motoneurons, provides a strong rationale for estimating PICs in both diaphragm and intercostal inspiratory motor units in humans to better understand inspiratory motor control.

In humans, the firing patterns of inspiratory motor units can be measured using decomposition of intramuscular electromyographic (EMG) recordings (Bolton et al., 1992; Hudson et al., 2025). The incrementing motoneuron activity during inspiration is probably a result of the well‐established ramp‐like firing pattern of phasically active excitatory bulbospinal neurons during inspiration (Monteau & Hilaire, 1991). However, other potentially non‐linear firing patterns have also been observed. For example, in the inspiratory human parasternal intercostal motor units, tonic firing during expiration in a proportion of motor units has been consistently shown (Butler et al., 2001; Gandevia et al., 2006; Hudson, Gandevia et al., 2011; Saboisky et al., 2007), which may be partly a result of the PIC contribution. Moreover, lower firing frequencies at motor unit derecruitment compared to recruitment are observed in both parasternal intercostal and diaphragmatic motor units during quiet and matched voluntary breaths (Hudson, Gandevia et al., 2011; Nguyen et al., 2019, 2020; Saboisky et al., 2007). This recruitment–derecruitment hysteresis could also be indicative of effects induced by calcium and sodium PICs. To test this hypothesis, the paired motor unit technique could potentially be used (Gorassini et al., 1998, 2002a, 2002b) on existing data during breathing to yield *in vivo* estimates of PIC contributions and further advance our understanding of the neural mechanisms regulating respiratory muscle control. This method is widely used to estimate PIC contribution in human limb motoneuron pools during ramp‐force contractions (Mesquita et al., 2024). These contractions are characterised by an increase followed by a decrease in net synaptic input and motor unit firing frequency. By using the firing frequency of a lower‐threshold ‘control’ motor unit as a proxy for net synaptic input, the difference in input level at the recruitment and derecruitment of a higher‐threshold ‘test’ unit (Δ*F* score) provides an indirect measure of PIC contribution to its firing prolongation (i.e. recruitment–derecruitment hysteresis). This approach has been validated by intracellular direct measurements of PICs in animal models (Bennett, Li, Harvey et al., 2001) and by computer simulations, which indicate that ΔF scores exceeding 1 Hz require a significant level of PIC activity generated by dendritic channels (Powers & Heckman, 2015). In addition, high levels of reliability for this method have also been shown in human data (Lapole et al., 2024). Rather than measure respiratory motor unit activity during ramped isometric contractions such as those commonly used to test the contribution of PICs in limb motor units, we investigated the evidence for PICs in respiratory motoneurons during their typical firing behaviour (i.e. during breathing). Importantly, inspiratory motor units generally display a rise‐and‐fall firing pattern over the course of inspiration (Butler et al., 2025). If this pattern is accompanied by a correlated trajectory between lower‐threshold control units and higher‐threshold test units, as observed in limb motor units, it would indicate that changes in the firing of the control unit reflect changes in synaptic input to the test unit, thereby enhancing the applicability of the ΔF technique to respiratory motoneurons and supporting its use for estimating PIC contributions *in vivo*.

In addition, it is difficult to postulate whether the magnitude of the contribution of PICs to inspiratory motor unit activity varies depending on whether these skeletal respiratory muscles are activated via bulbospinal (automatic) pathways or corticospinal (voluntary) pathways. On the one hand, greater serotonin release could occur during voluntary motor activity (Jacobs et al., 2002; Veasey et al., 1995) and enhance PIC activity (Hounsgaard et al., 1988). Conversely, activation of inhibitory spinal interneurons during both voluntary inspiration and expiration may lead to a concomitant increase in inhibition of inspiratory motoneurons (Andersen & Sears, 1970; Merrill & Fedorko, 1984). Because PICs in limb motoneurons are highly sensitive to inhibitory inputs (Heckman et al., 2008; Mesquita et al., 2022, 2024; Orssatto et al., 2022), this could attenuate their contribution.

Thus, the present study aimed to examine whether motor unit activity recorded from major human inspiratory muscles exhibits firing patterns indicative of prolongation of firing induced by motoneuronal PICs, and whether this potential PIC contribution differs between quiet (automatic) and voluntary breathing conditions. Understanding neural motor control of the inspiratory motoneurons is the foundation for developing therapeutic approaches for functional recovery of ventilatory and non‐ventilatory behaviours. Establishing whether PICs contribute to inspiratory motoneuron firing is essential for a mechanistic understanding of the neural motor control of respiratory motoneurons. Such knowledge would inform whether interventions known to modulate PIC activity in human spinal motoneurons are expected to be effective for targeting motor output during ventilatory and non‐ventilatory behaviours.

## Methods

Diaphragm (Experiment 1; Nguyen et al. (2019)) and parasternal intercostal motor unit data (Experiment 2; Hudson, Gandevia et al., (2011)) derived from these experiments have been published previously. In the present study, identified motor unit firings were reanalysed to estimate the contribution of persistent inward currents to motoneuron firing.

### Participants and ethical approval

In Experiments 1 and 2, respectively, seven healthy adults aged 23–26 years (six males and one female) and five healthy adults aged 30–58 years (five males) were studied. Participants had no history of neurological or pulmonary disorders. Participants were fully informed of any risks or discomforts associated with the procedures before giving their written informed consent to participate. The procedures were approved by the Human Research Ethics Committee of South Eastern Sydney Local Health District (Experiment 1: ethics approval number 16/357) and the Human Research Ethics Committee of the University of New South Wales (Experiment 2; ethics approval number 06151) and were performed in accordance with the *Declaration of Helsinki*, except for registration in a database (World Health Association, 2025).

### Procedures

In both experiments, participants visited the laboratory on one occasion. Participants sat on a chair, wore a nose clip and breathed through a mouthpiece connected to a pneumotachometer (Hans Rudolph, Inc., Shawnee, KS, USA) to measure airflow, which was also integrated to give inspiratory lung volume. End‐tidal CO_2_ was also measured (Normocap CO_2_ monitor; Datex, Helsinki, Finland).

Intramuscular EMG activity was recorded from the diaphragm in Experiment 1, and from the 1st, 3rd and 5th parasternal intercostal muscles in Experiment 2 to identify motor unit activity. A monopolar needle electrode (length, 50 mm; diameter, 0.46 mm; recording area, 0.34‐mm^2^; DMG50; Natus Medical Incorporated, Middleton, WI, USA) was used. In Experiment 1, it was inserted through the 7th or 8th intercostal space close to the mid‐clavicular line into the right costal diaphragm. In Experiment 2, it was inserted in each interspace (1st, 3rd and 5th) close to the lateral sternal edge into the parasternal intercostal muscles on the right‐hand side. Prior to needle insertion, muscles were located by palpation and ultrasound (diaphragm: iU22; Philips, Eindhoven The Netherlands; parasternal intercostals: Acuson 128 XP; Acuson, Mountain View, CA, USA) with a linear probe. The ranges of estimated depth of the superficial surface of the muscles relative to the skin surface were 15–34 mm, 14–22 mm, 18–28 mm and 15–34 mm in the diaphragm, 1st, 3rd and 5th parasternal intercostals, respectively. A small dose (1–2 mL) of local anaesthetic (1 % lignocaine) was injected into the superficial subcutis prior to needle insertion in the diaphragm in all participants in Experiment 1, and prior to needle insertion in the parasternal intercostals in one participant in Experiment 2. A topical anaesthetic (EMLA cream; Aspen Pharmacare Australia Pty Ltd, St Leonards, NSW, Australia) was applied to the skin in the other participants of Experiment 2. The needle electrode was inserted into the skin and EMG was monitored using auditory feedback from a loudspeaker and visual feedback on an oscilloscope. When a site exhibited single motor unit activity with an acceptable signal‐to‐noise ratio based on visual assessment, the audio signal was turned off, and recordings were conducted without feedback to the participant.

In Experiment 1, after recording quiet, automatic breathing for ∼30 s, the needle was either repositioned to a new recording site nearby or removed from the diaphragm and reinserted at a slightly different angle. Diaphragm EMG recordings were sampled from 10 sites in each participant.

**Figure 1:**
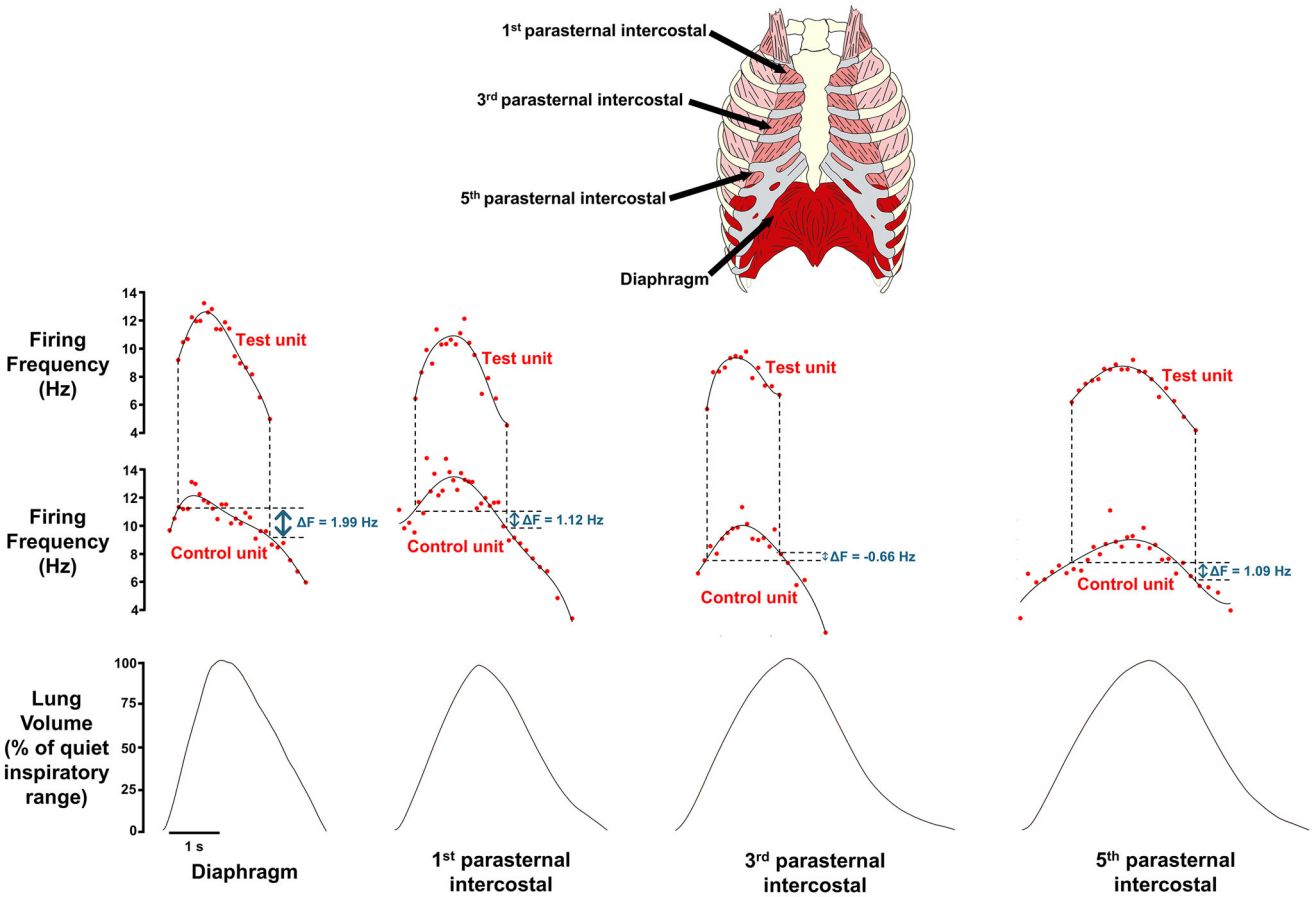
Examples of ∆*F* calculation used to estimate persistent inward currents (PICs) in different inspiratory muscles Representative paired motor unit recordings are shown for the diaphragm and the 1st, 3rd and 5th parasternal intercostal muscles, along with a schematic indicating their anatomical locations. For each muscle, the instantaneous (red dots) and smoothed (black line) firing frequencies of a test motor unit are displayed above those of a control unit, from which the Δ*F* value (blue) was derived. Δ*F* represents the difference in control unit smoothed firing rate at the recruitment and derecruitment of the test unit, providing an estimate of PIC contribution to the firing of the test unit. Lung volume is plotted below each trace to show the respiratory cycle occurring during the recorded motor unit activity. Schematic of respiratory muscles adapted from Hudson et al. (2019).

In Experiment 2, an initial 30‐s period of quiet, automatic breathing was recorded. During this period, participants did not receive visual feedback of lung volume, were not instructed on their breathing and were unaware that recordings were taking place. During the quiet breathing period, a researcher established a target lung volume for the voluntary breaths. The target increased through inspiration and was designed to match the mean inspiratory flow and tidal volume of the participant's quiet breathing. With the electrode in the same position, participants were provided with visual feedback of inspiratory lung volume and instructed to closely follow the target. There was no feedback of expiratory lung volume (see figure 1 in Hudson, Gandevia et al. (2011)). Participants typically needed six attempts (range: 3–20) to achieve three successful voluntary breaths that closely matched the target (as judged by a dedicated investigator). Once this was completed, the electrode was repositioned to a new recording site. The procedure was similar for each interspace, and EMG recordings were sampled from 6–10 sites in each interspace.

All signals were saved on a computer via a data acquisition interface (1401; Cambridge Electronic Design, Cambridge, UK) for subsequent analysis. EMG was amplified (10,000× to 20,000×), band‐pass filtered (53–3000 Hz) and sampled at 10,000 Hz, and airflow, volume and end‐tidal CO_2_ sampled at 1000 Hz.

### Data analysis

For each recording site, three sequential quiet breaths (Experiments 1 and 2) and three voluntary breaths that were well‐matched to the target of inspiratory lung volume (Experiment 2) were initially chosen for offline analysis. Individual motor unit activity was identified in these breaths in a commercial software package (Spike 2; Cambridge Electronic Design), as previously described (Gandevia et al., 1996). Briefly, trigger levels were manually set to identify spikes of an appropriate signal‐to‐noise ratio. Then, EMG data were sorted into motor unit templates based on the shape of their action potentials, which could be tracked between breaths. A customised script enabled the visual inspection of each motor unit template. All motor unit activity was visually inspected to verify automatic detection. Motor unit firings could be added or excluded as necessary to correct for false positives or missed discharges based on comparison of the raw EMG, motor unit templates, and the instantaneous firing frequency plots viewed simultaneously.

To conduct a paired motor unit analysis to estimate PIC contribution to prolongation of firing (Gorassini et al., 1998, 2002a, 2002b), breaths were included for analysis if at least two phasic motor units were identified (i.e. tonic motor units with inspiratory modulation of activity were not considered), with the requirement that at least one higher‐threshold motor unit was derecruited before a lower‐threshold motor unit. The smoothed firing rate of a lower‐threshold motor unit (control unit) was used to estimate the magnitude of net synaptic input at recruitment and derecruitment of a higher‐threshold motor unit (test unit). The difference between the recruitment and derecruitment inputs yielded the ∆*F* (change in frequency) value, which serves as an estimate of the contribution of PICs to firing prolongation (i.e. recruitment–derecruitment hysteresis). MATLAB (MathWorks, Natick, MA, USA) scripts and functions were used to fit instantaneous motor unit firing frequencies with polynomial functions. Preferentially, a 5th degree polynomial function was used. However, all polynomial fits were visually evaluated, and if edge effects were observed at motor unit recruitment or derecruitment with a 5th degree polynomial (i.e. a clear mismatch between the change in the smoothed and instantaneous firing rates), a 4th degree polynomial was used instead. If mismatches persisted with a 4th degree polynomial, the motor unit from that specific breath was excluded from subsequent analyses (Mesquita et al., 2022, 2023). A 4th degree polynomial could only be fitted to motor units with at least five data points of instantaneous firing frequency. Consequently, motor units with fewer than six firing events were also excluded from further analysis.

To identify suitable motor unit pairs for analysis, in addition to the test unit being recruited after and derecruited before the control unit, criteria were adopted to test the assumption that the control unit was a suitable proxy for net synaptic input. The rate‐to‐rate correlation coefficients (*r*) between the smoothed firing rate polynomials of the test and control units (2000 data points per second) had to be ≥ 0.7 (Stephenson & Maluf, 2011). Moreover, a saturation criterion was used to ensure that the control unit increased its firing rate by at least 0.5 Hz after the recruitment of test unit (Stephenson & Maluf, 2011).

A repeated measures approach involving up to three breaths was employed to estimate the contribution of PIC activity to the firing of the test units (Mesquita et al., 2022). First, ∆*F* values were calculated for all possible motor unit pairs within each breath. Then, a merged set of pairs were identified; if a specific pair was identified across multiple breaths, the average Δ*F* was calculated. For each test unit, Δ*F* values were determined by averaging values obtained from pairings with multiple suitable control units, as previously conducted (Trajano et al., 2020). Recruitment and derecruitment thresholds, as well as peak and average smoothed firing rates were also averaged across breaths. LabChart macros (ADInstruments, Dunedin, New Zealand) were used to quantify onset and offset parameters. The identification of suitable pairs and calculation of Δ*F* values was conducted in Excel (Microsoft Corp., Redmond, WA, USA).

An inspiration‐to‐expiration motor unit firing duration ratio was also calculated to assess asymmetry in motor unit firing during the inspiratory and expiratory phases, which were identified from lung volume using a technique adapted from the ‘self sustained firing duration’ reported in Afsharipour et al. (2020). The duration ratio was determined by subtracting the duration of motor unit firing during expiration from the duration of motor unit firing during inspiration, and then dividing the difference by the total duration of motor unit firing. Duration ratios were averaged across breaths. The resulting values ranged from –1 to 1, with a value of 1 indicating motor units active only during inspiration. A value of –1 would have corresponded to exclusive activity during expiration. This symmetric index has been used in previous studies (Afsharipour et al., 2020; Hassan et al., 2021; Jenz et al., 2023; Mohammadalinejad et al., 2024) and allows direct comparison with existing literature. To provide a more intuitive measure, we also expressed motor unit activity as the proportion (percentage) of total firing time occurring during inspiration. Although mathematically related, the two metrics emphasise comparability with prior work and interpretability, respectively.

### Statistical analysis

Statistical analyses were conducted in R, version 4.2.3 (R Foundation, Vienna, Austria), using RStudio environment (version 2023.06.0), except if indicated otherwise. *P* < 0.05 was considered statistically significant. Separate linear mixed‐effect models were computed to examine whether there were differences between muscles in ∆F, peak smoothed firing rate, average smoothed firing rate, recruitment threshold, firing rate range (peak smoothed firing rate minus minimum smoothed firing rate) and duration ratios (lmerTest package; Bates et al., 2015; Kuznetsova et al., 2017). The linear mixed‐effect model for firing rate range only included data from control units given the importance of their firing rate modulation for Δ*F* calculation. These outcome variables were analysed with a random intercept (parallel slopes) model, which accounted for the nested structure of the data. Specifically, models included ‘muscle’ as a fixed effect, and ´participant´ as a random effect (*Variable ∼ muscle +* (*1 | Participant*)). Residuals were plotted against fitted values to assess whether variance was consistent across the fitted range and Q–Q plot inspection was used to assess the assumption of normality of residuals. Estimated marginal means [with 95% confidence intervals (CI)] were quantified (emmeans package; Lenth et al., 2017). A false discovery rate analysis was used in post‐hoc comparisons to minimise the risk of type I error resulting from multiple pairwise comparisons.

To test whether ∆*F* values were greater than 0 Hz, linear mixed‐effects models were used which included a fixed effect for the intercept and a random effect for the participant (*∆F ∼ 1 + *(*1 | Participant*)). This allowed the examination of whether the intercept, which represents the overall mean ∆*F* value, differed from zero. Then, two‐tailed *P* values were halved to perform one‐tailed tests to examine whether ∆*F* values were significantly greater than zero. To test whether ∆*F* values were greater than 1 Hz, a transformed variable (∆*F* – 1) was created by subtracting 1 from ∆*F* values. The model was then fitted as described above. This was conducted given that a simulation study (Powers & Heckman, 2015) has indicated that ∆*F* values ≥1 Hz largely reflected PICs generated by dendritic channels. As some muscle‐specific mixed models produced singular fits, indicating that the participant‐level random‐effect variance was estimated as zero, sensitivity analyses were also performed in which ΔF values were first averaged within each participant and muscle. Intercept‐only linear models were then fitted separately for each muscle to these participant‐level means to test whether mean ΔF values were greater than 0 Hz and 1 Hz (*mean ΔF* ∼ *1*).

Separate linear mixed‐effect models were also computed to examine whether there were differences in parasternal intercostal ∆*F* values or duration ratios between quiet and voluntary breaths. Models included ‘condition’ as a fixed effect, and ‘motor unit’ nested in ‘participant’ as random effects (*Variable ∼ condition + *(*1 | Participant/Motor Unit*)) when pairs and motor units were tracked between conditions. Tracking the same pairs between quiet and voluntary conditions did not permit a thorough examination of potential differences in ∆*F* for the 3rd and 5th parasternal intercostal muscles. Specifically, only six test units (from three participants) were identified for the 3rd, and two test units (from two participants) identified for the 5th interspaces. Thus, an additional analysis was conducted (*Variable ∼ muscle + *(*1 | Participant*)), considering all test units identified in quiet and voluntary conditions (i.e. tracking of pairs or test units not considered). This approach enabled an examination of potential differences in the 3rd parasternal intercostal muscle but remained limited for the 5th parasternal intercostal muscle, with only 3 test units identified during voluntary breathing.

*Z* tests for independent proportions were conducted using SPSS Statistics software, version 29 (IBM Corp., Armonk, NY, USA) to examine whether the proportion of motor units with a duration ratio of 1 differed across muscles.

Finally, exploratory repeated measures correlations were also computed (rmcorr package; Bakdash & Marusich, 2017) to investigate associations between ∆*F* values of test units and their recruitment thresholds and peak smoothed firing rates, as well as whether the ∆*F* of all computed pairs was associated with the time between recruitments of control and test units. These correlations were computed for all muscles except the 5th parasternal intercostal muscle, due to the lower number of observations.

## Results

In total, 248 breaths were examined across muscles and conditions. Descriptive statistics related to number of breaths per muscle and motor unit analysis are presented in Table 1.

**Table 1:** Descriptive statistics for number of breaths and motor unit data

	Quiet breathing				Voluntary breathing		
	Diaphragm	1st PSIC	3rd PSIC	5th PSIC	1st PSIC	3rd PSIC	5th PSIC
Breaths	48	47	38	27	34	38	16
Motor units for analysis (range per breath)	47 (2–3)	47 (2–6)	47 (2–6)	28 (2–4)	37 (2–6)	44 (2–6)	23 (2–4)
% 5th degree polynomials	84.0	68.1	78.9	74.1	76.3	61.1	61.1
% 4th degree polynomials	16.0	31.9	21.1	26.0	23.7	38.9	38.9
% correlated motor unit pairs with *r* > 0.7	71.2	78.0	52.0	65.0	81.0	53.4	66.7

*Note*: Breaths, number of individual breaths examined with at least two phasic motor units. Motor units for analysis (range per breath): total number of motor units (and range per breath) used for further analysis (i.e. that could be fitted with either a 5th or 4th degree polynomial in breaths where at least one other motor unit could also be fitted with a polynomial function). Details on the number of participants, motor units and test units utilised in each statistical test are provided in the in‐text descriptions in the Results section. % 5th/4th‐degree polynomials: percentage of motor units fitted with a 5th or a 4th degree polynomial function. % correlated motor unit pairs with *r* > 0.7: percentage of examined motor unit pairs with a Pearson's correlation coefficient (*r*) greater than 0.7. PSIC: parasternal intercostal muscle.

### Quiet breathing

**Figure 2:**
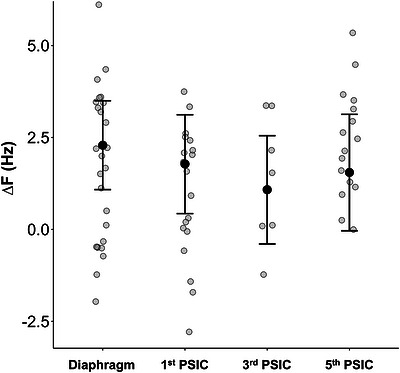
Estimates of persistent inward currents (∆*F*) during quiet breathing for each muscle Showing the ∆*F* scores of individual test units across the four muscles: 1st parasternal intercostal muscle (PSIC) with 26 test units from five participants, 3rd PSIC with 19 test units from three participants, 5th PSIC with eight test units from three participants and diaphragm with 16 test units from six participants. Estimated marginal means are represented by black circles with 95% confidence intervals indicated. The linear mixed‐effects model did not reveal significant differences between muscles. Diaphragm ∆*F* scores were significantly greater than 1 Hz, and all muscles had ∆*F* scores significantly greater than 0 Hz.

Representative paired motor unit recordings for each muscle are shown in Fig. 1. Significant differences were not detected (*F*_23,3_ = 0.91, *P* = 0.449) between muscles on ∆*F* scores, and the estimated Δ*F* mean scores (95% confidence intervals) were: diaphragm: 2.29 Hz (1.08–3.5); 1st parasternal intercostal: 1.78 Hz (0.43–3.12); 3rd: 1.08 Hz (–0.39 to 2.55); 5th: 1.55 Hz (–0.04 to 3.13) (Fig. 2). Diaphragm was the only muscle with a mean ∆*F* score significantly greater than 1 Hz [diaphragm: *t*_15_ = 3.64, *P* = 0.001, *n* = 6 participants (16 test units); 1st parasternal intercostal: *P* = 0.170, *n* = 5 (26 test units); 3rd: *P* = 0.489, *n* = 3 (19 test units); 5th: *P* = 0.302, *n* = 3 (8 test units)]. All muscles exhibited mean ∆F scores significantly greater than 0 Hz (diaphragm: *P* < 0.001; 1st parasternal intercostal: *P* = 0.041; 3rd: *P* = 0.012; 5th: *P* = 0.028). However, because the random‐effect variance collapsed to zero for diaphragm, 3rd, and 5th parasternal intercostals, but not for the 1st parasternal intercostal, the main mixed‐model analysis effectively used test‐unit‐level degrees of freedom for diaphragm, 3rd, and 5th, while the 1st parasternal intercostal model retained participant‐level clustering. We therefore repeated the analysis using participant‐level mean ΔF values; this sensitivity analysis retained the conclusion that only diaphragm was significantly greater than 1 Hz (diaphgragm: *P =* 0.004; 1st: *P =* 0.193; 3rd: *P =* 0.346; 5th: *P =* 0.504) and showed that only diaphragm and 1st parasternal intercostal had mean ΔF values significantly greater than 0 Hz (diaphragm: *P <* 0.001; 1st: *P =* 0.046), whereas 3rd and 5th did not (3rd: *P =* 0.067; 5th: *P =* 0.089).

The average time differences between the recruitment of control and test units were 0.24 ± 0.19 s for diaphragm, 0.25 ± 0.23 s for the 1st, 0.36 ± 0.34 s for the 3rd and 0.47 ± 0.44 s for the 5th parasternal intercostal muscles. There were no significant correlations between the recruitment time differences and ∆F scores for any of the muscles (*r*_rm_ = –0.08 to 0.27, *P* = 0.130–0.770).

When examining differences in the proportion of motor units that continued to fire into the expiratory phase (duration ratio < 1), the diaphragm exhibited a significantly higher proportion (85.1% of 47 MUs) than the 1st (48.9% of 47 MUs, *P* < 0.001) and 3rd (63.8% of 47 MUs, *P = *0.018) parasternal intercostal muscles, but not different to the 5th (71.4% of 28 MUs, *P = *0.152). Estimated marginal means (95% CI) of duration ratios for each muscle were 0.46 (0.21–0.71) for diaphragm, 0.83 (0.56–1.00) for the 1st parasternal intercostal, 0.88 (0.60–1.00) for the 3rd and 0.61 (0.34–0.89) for the 5th. These values correspond to estimated proportions of total motor unit firing time occurring during inspiration of 73% (61–86) for the diaphragm, 91.5% (78–100) for the 1st parasternal intercostal, 94% (80–100) for the 3rd and 80.5% (67–95) for the 5th. A significant effect of muscle was observed (*F*_21,3_ = 7.36, *P* = 0.001), with *post hoc* comparisons revealing that the 5th parasternal intercostal muscle exhibited significantly lower duration ratios compared to both the 1st (*P = *0.003) and the 3rd (*P* < 0.001) parasternal intercostal muscles. No other statistically significant differences were detected by the linear mixed model, despite the lower absolute estimated marginal mean value for diaphragm MU duration ratios. Estimated marginal means and confidence intervals for other secondary variables (i.e. peak, averaged and ranges of smoothed firing rates, and recruitment thresholds), as well as indication of significant differences between muscles, can be found in Table 2.

**Table 2:** Motor unit firing rates and recruitment thresholds during quiet breathing

Muscle	Peak smoothed firing rate (Hz)	Averaged smoothed firing rate (Hz)	Smoothed firing rate range (Hz) (control units only)	Recruitment threshold (%)
Diaphragm	14.5 (13.0–16.1)^a^	11.8 (10.5–13.1)^a^	9.3 (8.04–10.64)^a^	28.3 (13.3–43.4)^a,b^
1st PSIC	13.8 (12.1–15.5)^a^	11.9 (10.4–13.3)^a^	8.01 (6.71–9.31)^a^	13.7 (0–30.2)^a^
3rd PSIC	10.6 (8.9–12.4)^b^	9.0 (7.5–10.5)^b^	6.03 (4.61–7.45)^b^	23.8 (7.0–40.6)^a,b^
5th PSIC	10.5 (8.8–12.3)^b^	8.8 (7.3–10.3)^b^	6.32 (4.87–7.77)^b^	28.7 (11.7–45.6)^b^

*Note*: Peak and averaged firing rates and firing rate ranges were calculated from smoothed firing data obtained via polynomial functions, whereas recruitment thresholds were determined based on lung volume (with the peak of inspiratory lung volume set as 100%). Estimated marginal means (95% confidence intervals) are indicated. Superscript letters indicate differences between muscles: muscles sharing the same letter are not significantly different, and muscles with different letters are significantly different. Muscles with combined letters (i.e. ‘a,b’) are not significantly different from muscles with ‘a’ or ‘b’.

### Voluntary breathing

**Figure 3:**
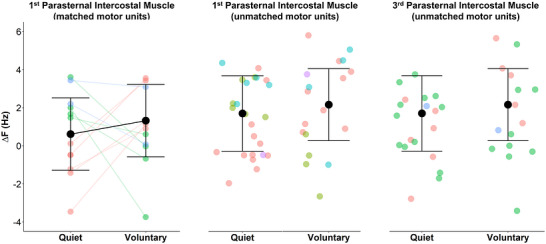
Comparison of estimates of persistent inward currents (∆*F*) between quiet and voluntary breathing Left: illustrating changes in ∆*F* scores between quiet [0.62 Hz; 95% confidence interval (CI) = –1.29 to 2.52] and voluntary (1.32 Hz; 95% CI = –0.58 to 3.22) breathing in the 1st parasternal intercostal muscle, where the same motor unit pairs were tracked across conditions (12 test units from three participants). The central panel presents an unmatched motor unit comparison, displaying ∆*F* scores from the 1st parasternal intercostal muscle during quiet (25 test units; 1.70 Hz; 95% CI = –0.28 to 3.68) *vs*. voluntary breathing (18 test units; 2.16 Hz; 95% CI = 0.27–4.05) from four participants, without tracking the test units between conditions. The right panel also shows an unmatched comparison of ∆F scores between quiet (1.01 Hz; 95% CI = –1.10 to 3.13) and voluntary (1.66 Hz; 95% CI = 0.00–3.33) conditions, but from the 3rd parasternal intercostal muscle from three participants. In all panels, datapoints are colour‐coded by participant, and estimated marginal means are represented by black circles with 95% CI. No significant differences between conditions were found.

When tracking the same motor unit pairs between quiet and voluntary breathing, ∆*F* scores from 12 test units (*n* = 3) of the 1st parasternal intercostal muscle were compared between conditions (Fig. 3, left), without evidence of a significant difference (*F*_22,1_ = 0.54, *P = *0.469). The unmatched analysis (i.e. tracking of the same test units was not considered in the model) allowed for the comparison of ∆*F* scores between quiet and voluntary breathing from 25 and 18 test units (*n* = 4) for the 1st parasternal intercostal (Fig. 3, central) and from 19 and 15 test units (*n* = 3) for the 3rd parasternal intercostal muscle (Fig. 3, right). No significant differences were observed for either the 1st (*F*_38,1_ = 0.51, *P = *0.478) or the 3rd (*F*_32,1_ = 0.79, *P = *0.340) parasternal intercostals. A robust comparison of ∆F scores between quiet and voluntary breathing remained unfeasible for the 5th parasternal intercostal muscle, as only eight and three test units, respectively, from two participants could be considered.

In voluntary breaths, the average time differences between the recruitment of control and test units included in the unmatched analysis were 0.16 ± 0.22 s for the 1st, and 0.36 ± 0.22 s for the 3rd parasternal intercostal muscles. No significant correlations between the recruitment time differences and ∆*F* scores were found for either muscle (1st: *r*_rm_ = 0.16, *P* = 0.307; 3rd: *r*_rm_ = 0.28, *P* = 0.121).

To examine duration ratios, a matched motor unit analysis was conducted. The percentage of motor units that were tracked from quiet to voluntary breathing were 72.3%, 80.1% and 82.6% in the 1st, 3rd and 5th parasternal intercostal muscles, respectively. In the 1st parasternal intercostal, there was no evidence of significant differences of duration ratios (*F*_33,1_ = 1.49, *P = *0.232) between quiet (0.82; 95% CI = 0.62–1.00; proportion of time spent in inspiration: 91%; 95% CI = 81–100) and voluntary breathing (0.87; 95% CI = 0.67–1.00; 94%; 95% CI = 84–100). However, duration ratios were significantly higher in voluntary breathing for both the 3rd (0.68; 95% CI = 0.40–0.97 *vs*. 0.84; 95% CI = 0.56–1.00; 84%; 95% CI = 78–100 *vs*. 92%; 95% CI = 0.78–100; *F*_37,1_ = 10.23, *P = *0.003) and the 5th parasternal intercostals (0.59; 95% CI = –0.15 to 1.00 *vs*. 0.80; 95% CI = 0.06–1.00; 80%; 95% CI = 43–100 *vs*. 90% (95% CI = 53–100; *F*_18,1_ = 11.84, *P = *0.003), suggesting an earlier recruitment of motor unit firing activity relative to the onset of inspiration.

## Discussion

To the best of our knowledge, the present study is the first to estimate the contribution of PICs to respiratory motoneuron firing in humans. This estimation was performed using the well‐established paired motor unit technique, which quantifies recruitment–derecruitment firing hysteresis (Gorassini et al., 1998, 2002a, 2002b) and has been extensively applied to estimate PICs in human limb‐muscle motoneuron pools (Mesquita et al., 2024). The observed magnitude of firing prolongation (i.e. recruitment–derecruitment hysteresis quantified via Δ*F* scores) in diaphragmatic motor units during quiet breathing is indicative of an involvement of motoneuronal PICs. By contrast, the lower and more variable firing prolongation in the 1st, 3rd and 5th parasternal intercostal motor units during quiet or voluntary breathing does not support a consistent contribution of PICs in these motoneuron pools. These findings enhance our understanding of the neural control of human inspiratory muscles and motivate further investigations into the mechanisms underlying differences in neuromodulation and firing prolongation across respiratory motoneuron pools, as well as the functional implications of such differences.

### Evidence of PIC contribution to diaphragmatic motoneuron firing

To examine whether the firing patterns of obligatory inspiratory muscles exhibited firing prolongation with magnitudes that were indicative of PIC involvement, we tested whether estimates of PIC‐induced firing prolongation (i.e. ∆*F* scores) were significantly greater than 1 Hz, a threshold suggested by a simulation study by Powers & Heckman (2015). Our data showed that this criterion was met for diaphragmatic motor units but not for parasternal intercostal motor units. Although previous studies in limb motoneuron pools have not formally conducted this type of analysis to the best of our knowledge, the vast majority of previously reported ∆*F* scores were probably significantly greater than 1 Hz. For example, on average, tibialis anterior, gastrocnemius medialis and soleus exhibit ∆*F* scores of 4.60, 3.15 and 2.72 Hz, respectively, according to a recent review (Mesquita et al., 2024). Future studies could usefully replicate this analytical approach as an objective method to assess whether motor unit firing patterns suggest PIC‐induced firing prolongation.

The observation of ∆*F* scores in diaphragmatic motor units with a magnitude indicative of PIC‐induced firing prolongation (i.e. mean 2.29 Hz) contrasts with the suggested absence of PIC involvement in cat phrenic inspiratory motoneurons (Enríquez Denton et al., 2012). However, there are several methodological differences between the studies. First, Enríquez Denton et al. (2012) assessed voltage‐dependent amplification of synaptic inputs (i.e. the increase in peak depolarisation amplitude during inspiration at more depolarised membrane potentials), whereas the present study assessed recruitment–derecruitment hysteresis (∆*F* in human studies or ∆*I*, change in injected current, for cellular studies). In motoneuron firing, synaptic input amplification is expected to lead to the non‐linear behaviours of firing acceleration on recruitment and saturation (Beauchamp et al., 2023; Binder et al., 2020; Chardon et al., 2023; Heckman et al., 2008; Mesquita et al., 2024; Powers & Heckman, 2017; Zero et al., 2022), whereas firing prolongation is believed to have a different underlying biophysical mechanism (Binder et al., 2020; Shapiro & Lee, 2007). Thus, PICs could potentially contribute to firing prolongation in diaphragmatic motoneurons without necessarily affecting synaptic input amplification. Second, the electrodes used in Enríquez Denton et al. (2012) contained the local anaesthetic derivative QX‐314, which could have blocked PIC activity (Lee & Heckman, 1999), potentially limiting detection of PIC involvement. Third, the present study was conducted in humans as opposed to cats, raising the possibility that the existence and contribution of PIC channels to inspiratory motoneuron firing may differ between species. Fourth, different populations of diaphragmatic motoneurons were probably examined in the two studies. Although it remains unclear whether PIC magnitude varies with motoneuron size (Mesquita et al., 2024), the present study probably identified smaller, lower‐threshold diaphragmatic motor units during quiet breathing (Seven et al., 2013; Sieck & Fournier, 1989). By contrast, the sample of phrenic motoneurons identified in Enríquez Denton et al. (2012) was biased towards larger motoneurons. Finally, it cannot be ruled out that mechanisms independent of PICs could have contributed to positive ∆*F* scores, including slow inactivation of Kv1.2 channels in the axon initial segment (Bos et al., 2018) and spike frequency adaptation (Revill & Fuglevand, 2011). Although NMDA receptor activity has not been directly linked to recruitment–derecruitment hysteresis, the proposed role of NMDA‐mediated currents in synaptic input amplification in cat phrenic motoneurons (Enríquez Denton et al., 2012), and the capacity of NMDA to induce membrane potential oscillations that drive rhythmic motoneuron firing in adult rodents (Manuel et al., 2012), suggests that a potential contribution of NMDA receptors to the Δ*F* scores observed here cannot be entirely excluded.

### Variability and potential absence of significant PIC contribution in parasternal intercostal motoneurons

The lower and more variable magnitudes of firing prolongation observed in the 1st, 3rd and 5th parasternal intercostal motor units during quiet breathing and the absence of significant differences in matched voluntary breaths were unexpected and suggest that PICs may not consistently contribute to firing in these motoneuron pools. Alternatively, the lack of ∆*F* scores > 1 Hz at the group level could reflect variability in the number of PIC channels, their activity, or a variable influence of descending monoaminergic inputs across the motoneuron pool (De Troyer et al., 2005; Sharples & Miles, 2021; Zhan et al., 2000). Another potential explanation is a non‐uniform distribution of inhibitory inputs or varying levels of sensitivity to inhibition across the motoneuron pool (De Troyer et al., 2005; Mesquita et al., 2020). For example, in certain motoneurons, PICs could actually contribute to the initiation or acceleration of firing, but the influence of inhibitory inputs before motoneuron derecruitment could make the detection of recruitment–derecruitment hysteresis less probable. Finally, we cannot rule out the possibility that the variability and potential absence of significant PIC contribution in parasternal intercostal motoneurons may be influenced by age rather than muscle‐specific factors, given the older age of the parasternal intercostal cohort (30–58 years) compared to the diaphragm cohort (23–26 years). However, this possibility is not supported by recent evidence (Mohammadalinejad et al., 2024) showing no significant differences in ΔF tibialis anterior motor units between young (18–28 years) and middle‐aged adults (32–53 years).

In the present study, we observed no significant differences in ΔF between quiet and voluntary breaths. This indicates that altering the source of descending drive to inspiratory motor units (i.e. bulbospinal *vs*. corticospinal), while matching voluntary output, did not measurably alter the estimated contribution of PICs to motoneuron firing. Although an increase in voluntary drive could be associated with changes in monoaminergic input (Jacobs et al., 2002; Veasey et al., 1995) or spinal inhibitory activity (Andersen & Sears, 1970; Merrill & Fedorko, 1984), both of which are known to modulate PIC expression (Heckman et al., 2008; Hounsgaard et al., 1988; Mesquita et al., 2022, 2024; Orssatto et al., 2022), these mechanisms were not directly assessed in the present study. Therefore, the absence of differences in Δ*F* may reflect that these potential modulatory influences associated with voluntary drive were minimal. Alternatively, voluntary breathing may have had a concurrent facilitatory and inhibitory effect on PICs, resulting in no net change in firing prolongation. Finally, given the low and inconsistent magnitude of the estimated PIC‐associated firing prolongation in parasternal intercostal motor units, another possibility is that a floor effect may have constrained the detectable range of PIC modulation.

### Methodological considerations in estimating PICs in respiratory motoneurons

In the present study, ∆*F* scores in both diaphragmatic and parasternal motor units were lower than most ∆*F* scores reported in limb‐innervating motor units (Mesquita et al., 2024). This suggests the possibility that PICs play a less prominent role in firing prolongation in inspiratory motoneurons compared to limb motoneurons. However, meaningful comparisons require consideration of key methodological and physiological differences between these systems.

In animal models, PICs can be quantified using triangular current injections, with the difference in injected current at motoneuron recruitment and derecruitment (i.e. Δ*I*) serving as a direct measurement of PIC‐induced recruitment–derecruitment hysteresis (Bennett, Li, Siu et al., 2001). In humans, where such direct current manipulation is not feasible, Δ*F* (i.e. change in firing frequency of the control unit that serves as a proxy of net synaptic input) provides an indirect estimate of Δ*I*. Although motor unit firing rates in limb muscles tend to scale more symmetrically with force during isometric ramp contractions (Negro et al., 2009), inspiratory motor units show less direct correspondence between firing rate modulation and mechanical output, with motor unit firing typically timed with the central respiratory drive potential peaking during inspiration and declining before its end, because expiration is largely passive in humans (Butler et al., 2025). Nonetheless, Δ*F* calculation is not based on force or flow symmetry, but on the trajectory of the control unit's smoothed firing rate, which typically increases and then decreases during the inspiratory effort. In the present study, the mean firing rate ranges of control units in the diaphragm and 1st parasternal intercostal muscles resembled those of tibialis anterior motor units, whereas the 3rd and 5th parasternal intercostals were comparable to soleus motor unit ranges, consistent with those reported by McPherson et al. (2025) during triangular isometric contractions at 20% of peak force in healthy subjects. A rise‐and‐fall pattern with high rate‐to‐rate correlation between test and control units suggests an increase followed by a decrease in net synaptic input, allowing for a meaningful Δ*F* quantification even in the absence of mechanical symmetry. Similar dissociations between motor unit firing and contraction intensity have been reported in limb (e.g. gastrocnemius medialis; Mesquita et al., 2022, 2023) and axial muscles (trapezius; Stephenson & Maluf, 2010), further supporting the broader applicability of the Δ*F* method. Although care is warranted in interpreting Δ*F* scores across motoneuron pools with very distinct cortical and subcortical inputs, its use in inspiratory motor units remains conceptually justified.

An important methodological distinction is that ∆*F* scores in limb‐innervating muscles are typically calculated during ∼20 s ramp contractions, whereas the breaths analysed in the present study had durations of only ∼2 s during inspiration and ∼3 s during expiration, with peak firing occurring prior to end inspiration. The kinetics of full PIC activation are relatively slow (i.e. 1–2 s; Binder et al., 2020) compared to other voltage‐gated ion channels (i.e. 1–10 ms; Catterall, 1995), probably because of the time required for calcium‐dependent facilitation to enhance the opening of the calcium PIC channels (Binder et al., 2020). Thus, it cannot be ruled out that calcium PICs were not fully activated during each breath, which may have contributed to the relatively lower ∆F scores observed. This idea is supported by prior findings where test motor units that were active for a short period before peak net synaptic input tended to exhibit lower Δ*F* (Afsharipour et al., 2020) or Δ*I* scores (Li et al., 2004). Alternatively, although the respiratory motor units under examination in the present study exhibited clear phasic activity, it remains uncertain whether PICs, once activated, fully deactivate between breaths, particularly if inhibitory input during the expiration phase is insufficient to terminate them. If PICs persist in a subthreshold state across respiratory cycles, they may continue to influence motoneuron intrinsic excitability between each motoneuron derecruitment and subsequent recruitment.

This nuance also highlights another methodological aspect of the Δ*F* quantification. Estimating PIC contributions during functionally relevant, short‐duration breathing tasks precluded the use of a minimum time interval (commonly 1 s; Mesquita et al., 2024) between the recruitment of control and test units. This interval is typically employed to ensure that PICs in control units have had sufficient time to fully activate and thus serve as a valid surrogate of changes in net synaptic input. If PICs, once activated, persist in a subthreshold state between breaths rather than fully deactivating, the requirement for a minimum recruitment interval to ensure PIC activation could be less critical in respiratory motor units. Interestingly, our analyses found no significant associations between recruitment time differences and ∆*F* scores. Furthermore, it is unlikely that motor unit pairs were included in the analysis with control units that exhibited high degrees of secondary range firing (as a result of slow PIC activation) that persisted beyond the test unit's recruitment. This is because the rate‐to‐rate correlation of such pairs would probably be low, and we only included pairs with rate‐to‐rate correlation coefficients higher than 0.70. Although the proportion of included unit pairs satisfying this criterion (∼50–80% across muscles in the present study) is informative, this percentage has not been previously reported in studies on limb‐innervating motor units, precluding direct comparisons. Future studies could usefully compute ∆*F* values from inspiratory motor unit activity during volitionally prolonged breaths or isometric contractions. Extending breath duration may increase the possibility of full calcium PIC activation, potentially resulting in greater magnitudes of firing prolongation. Such examinations could provide deeper insights into whether the role of PICs in inspiratory motoneurons depends on the duration of inspiratory activity and would allow robust quantifications of other non‐linearities such as firing rate acceleration via computation of brace heights (Beauchamp et al., 2023) or consecutive linear fit functions (Afsharipour et al., 2020; Mohammadalinejad et al., 2024).

### Directions for future research

It has been extensively documented that inspiratory motor unit activity can persist into expiration during eupnea (Dutschmann et al., 2014; Gesell & White, 1938; Richter, 1982). This persistent activity during expiration has been referred to as post‐inspiratory activity and both afferent and descending pathways appear to be involved in its generation (Dutschmann et al., 2014). Could intrinsic motoneuron excitability also contribute to this phenomenon? In the present study, we quantified the proportion of individual motor unit activity occurring during expiration relative to inspiration using duration ratios. This metric was adapted from previous studies that quantified firing asymmetry between ascending and descending phases of ramp isometric contractions as a complementary variable in the estimation of PIC contribution to firing prolongation (Afsharipour et al., 2020; Hassan et al., 2021; Jenz et al., 2023; Mohammadalinejad et al., 2024). Interestingly, the proportion of motor units that continued to fire during expiration (i.e. duration ratio < 1) was higher in diaphragmatic motor units than in the 1st and 3rd parasternal intercostals. As an interesting supplementary finding, the computation of duration ratios for parasternal intercostal motor units in this study further underscores their rostro‐caudal gradient with differences in the timing and firing rates of motor unit activity during voluntary breathing, as previously reported (Hudson, Gandevia et al., 2011). Future studies in animal models utilising more precise quantification of PIC magnitudes could investigate the impact of blocking or enhancing PICs on the duration of post‐inspiratory activity and whether such an effect would have a functional relevance such as modulating expiratory airflow which could affect lung volumes and respiratory mechanics (O'Donnell & Laveneziana, 2006). In animal models, increased expiratory muscle activity has been observed during hypercapnia (Abdala et al., 2009; Flor et al., 2020; Molkov et al., 2010), a condition in which efficient CO_2_ elimination becomes crucial. Functionally, prolonged post‐inspiratory activity under such conditions could be counterproductive to the goal of augmenting expiratory airflow. Consistent with this notion, a marked decrease in the duration of post‐inspiratory diaphragm activity was recently reported in awake rats (Khurram et al., 2024). If PICs contribute to post‐inspiratory activity, it could be postulated that activation of inhibitory spinal interneurons during states of enhanced expiratory drive (Andersen & Sears, 1970; Merrill & Fedorko, 1984) may suppress PIC‐mediated inspiratory activity, thereby shortening its persistence into expiration. Conversely, intermittent hypercapnia could have the opposite effect, given its possible facilitatory effect in human inspiratory motoneuron pools (Mesquita, 2022; Welch et al., 2022). Future studies could usefully explore whether such state‐dependent modulations of post‐inspiratory activity are also present in humans and, if so, whether they are mediated by PICs.

Although the present study provides novel insights into the contribution of PICs to inspiratory motoneuron firing in humans, future research is needed to corroborate or refine our findings. For example, interventions that elicit inhibitory inputs onto motoneurons (Gomes et al., 2024; Mesquita et al., 2022; Orssatto et al., 2022; Pearcey et al., 2022), with ingestion of monoaminergic drugs (D'Amico et al., 2013; Goodlich et al., 2023; Mohammadalinejad et al., 2024; Udina et al., 2010), or studies examining ageing (Guo et al., 2024; Hassan et al., 2021; Mohammadalinejad et al., 2024; Orssatto, Borg et al., 2021) have consistently demonstrated modulation of PIC activity. If differences in estimates of PIC activity in inspiratory motoneurons are observed in future studies employing similar experimental approaches, it would further support the hypothesis that PICs can play an important modulatory role in respiratory motoneuron output. Moreover, given the predominantly male sample and limited cohort size, future studies with larger and sex‐balanced samples are required to confirm these findings and to determine whether sex‐related differences in PIC contribution to respiratory motoneuron firing are present. Although the only studies that have formally examined sex differences in limb motoneuron pools reported greater PIC estimates in females in most (Jenz et al., 2023; Lecce et al., 2025; Yacyshyn et al., 2025) but not all cases (Mesquita et al., 2022, 2023), it remains unknown whether sex differences occur in respiratory motoneurons.

Could PICs contribute to firing prolongation in parasternal intercostal muscles in other physiological contexts, such as during trunk rotations? Unlike the diaphragm (Hudson, Butler et al., 2011), the parasternal intercostal muscles significantly contribute to ipsilateral rotations, and their phasic inspiratory activity is greatly increased when breathing occurs during such rotations (Hudson, Butler et al., 2011; Hudson et al., 2010, 2017). This increased activity could be driven by greater descending excitatory inputs from the cortex or medulla, as well as afferent inputs (Hudson et al., 2010). As PIC channels are voltage‐gated (Li et al., 2004; Svirskis & Hounsgaard, 1997), increased excitatory drive could augment PIC‐induced non‐linearities, as demonstrated in cat motoneurons (Hultborn et al., 2003) and inferred in human motor unit recordings (Goodlich et al., 2023; Lapole et al., 2024; Mackay et al., 2023; Orssatto, Mackay et al., 2021). Future studies could explore whether PICs significantly contribute to parasternal motoneuron firing during non‐respiratory ipsilateral rotations and during inspiratory activity in the presence of such trunk rotations.

New methodological paradigms could also further enhance our understanding of PICs in respiratory motor output. For example, PICs were not estimated in motor units exhibiting tonic firing, particularly in parasternal intercostal muscles, because of the inability to compute ∆*F* scores or duration ratios using the established methodology developed in phasically active units (Gorassini et al., 1998, 2002a, 2002b). Since self‐sustained firing is a hallmark of PIC activity (Binder et al., 2020; Crone et al., 1988; Eken & Kiehn, 1989; Hounsgaard et al., 1988; Lee & Heckman, 1996; Schwindt & Crill, 1980), it remains to be explored whether the firing of these motor units is sustained by ongoing excitatory input or by strong intrinsic motoneuron excitability, such as subthreshold oscillations of sodium PICs, as demonstrated in chronic spinal rats (Li et al., 2004).

Furthermore, additional methodological approaches would also have to be developed to investigate *in vivo* whether the activity of PIC channels in respiratory motoneurons play a functional role in other behaviours such as sneezing, coughing, breath‐holds, trunk rotations, vocalisation, swallowing or vomiting.

Establishing whether PICs contribute to inspiratory motoneuron firing is an important step towards informing whether interventions known to modulate PIC activity in spinal motoneurons have the potential to be effective for targeting respiratory motor output. The mechanistic knowledge presented here could also motivate future examinations of whether altered intrinsic motoneuron excitability plays a role in compromised respiratory motor output in pathological conditions.

### Conclusions

The present data demonstrate that intrinsic motoneuron excitability contributes to the control of firing output in human respiratory motoneurons, although this contribution is heterogeneously expressed across inspiratory muscles. Our findings suggest motoneuronal PICs contribute significantly to the prolonged firing of diaphragmatic motor units during quiet breathing, whereas their role in the 1st, 3rd and 5th parasternal intercostal motor units is not as evident during both quiet and voluntary breaths. It can then be postulated that there are fundamental differences in the neuromodulatory control of human inspiratory muscles. Future research should further explore the mechanisms driving these variations in non‐linear firing patterns and their functional implications for respiratory control in health and disease.

## Additional information

## Competing interests

The authors declare that they have no competing interests.

## Author contributions

J.E.B., A.L.H. and S.C.G. conceived and designed the research and performed experiments. R.N.O.M. and A.L.H. analysed data. R.N.O.M. prepared figures and drafted the manuscript. All authors interpreted the results of experiments, as well as edited and revised the manuscript. All authors approved the final version of the manuscript submitted for publication and agreed to be accountable for all aspects of the work in ensuring that questions related to the accuracy or integrity of any part of the work are appropriately investigated and resolved. All persons designated as authors qualify for authorship, and all those who qualify for authorship are listed.

## Funding

This work was funded by the National Health and Medical Research Council (NHMRC, Australia). SCG and JEB are supported by NHMRC Fellowships.

## Supporting information


Peer Review History

## Data Availability

Individual data that support the findings of this study are available from the corresponding author upon request.
