# Supplementary material for: Estimation of persistent inward currents contribution to inspiratory motoneuron firing in humans
Source: J Physiol. 2026 Jul 22;604(15):6488–505. doi: 10.1113/JP290663 (PMC13423127; doi:10.1113/JP290663)
Supplement: Supplementary file 1 — Peer Review History [file TJP-604-6488-s001.pdf]

The Journal of Physiology JP

Estimation of persistent inward currents contribution to inspiratory motoneuron firing in humans

Ricardo N. O. Mesquita, Simon C Gandevia, Janet L Taylor, Anna L Hudson, and Jane Butler
DOI: 10.1113/JP290663

Corresponding author(s): Ricardo Mesquita (ricardo.mesquita@ki.se)

The following individual(s) involved in review of this submission have agreed to reveal their identity: Gary C. Sieck (Referee #1); Allison S. Hyngstrom (Referee #2)

Review Timeline:

Submission Date:	28-Nov-2025
Editorial Decision:	05-Feb-2026
Revision Received:	27-Apr-2026
Accepted:	15-May-2026

Senior Editor: Richard Carson

Reviewing Editor: Madeleine Lowery

Transaction Report:

(Note: With the exception of the correction of typographical or spelling errors that could be a source of ambiguity, letters and reports are not edited. Depending on transfer agreements, referee reports obtained elsewhere may or may not be included in this compilation. Referee reports are anonymous unless the Referee chooses to sign their reports.)

Re: JP-RP-2025-290663 "**Estimation of persistent inward currents contribution to inspiratory motoneuron firing in humans**" by Ricardo N. O. Mesquita, Simon C Gandevia, Janet L Taylor, Anna L Hudson, and Jane E Butler

Dear Dr Mesquita,

Thank you for submitting your manuscript to The Journal of Physiology. It has been assessed by a Reviewing Editor and by 2 expert referees and we are pleased to tell you that it is potentially acceptable for publication following satisfactory major revision.

Please advise your co-authors of this decision as soon as possible.

The referee reports are copied at the end of this email.

Please address all the points raised and incorporate all requested revisions or explain in your Response to Referees why a change has not been made. We hope you will find the comments helpful and that you will be able to return your revised manuscript within 2 months. If your article is NOT for a Special Issue, you may have 9 months to revise. If you require an extension, please contact journal staff: jp@physoc.org. Please note that this letter does not constitute a guarantee for acceptance of your revised manuscript.

Your revised manuscript should be submitted online using the link in your Author Tasks: Link Not Available. This link is accessible via your account as Corresponding Author; it is not available to your co-authors. If this presents a problem, please contact journal staff (jp@physoc.org). Image files from the previous version are retained on the system. Please ensure you replace or remove any files that are being revised.

If you do not wish to submit a revised version of your manuscript, you must inform our journal staff (jp@physoc.org) or reply to this email to request withdrawal. Please note that a manuscript must be formally withdrawn from the peer review process at one journal before it may be submitted to another journal.

TRANSPARENT PEER REVIEW POLICY: To improve the transparency of its peer review process, The Journal of Physiology publishes online as supporting information the peer review history of all articles accepted for publication. Readers will have access to decision letters, including Editors' comments and referee reports, for each version of the manuscript, as well as any author responses to peer review comments. Referees can decide whether or not they wish to be named on the peer review history document.

ABSTRACT FIGURES: Authors are expected to use The Journal's premium BioRender account to create/redraw their Abstract Figures. Information on how to access this account is here: https://physoc.onlinelibrary.wiley.com/journal/14697793/biorender-access.

This will enable Authors to create and download high-resolution figures. If authors have used the free BioRender service, they can use the instructions provided in the link above to download a high-resolution version suitable for publication.

The link provided should only be used for the purposes of this submission. Authors will be charged for figures created on this account if they are not related to this manuscript submission.

LANGUAGE EDITING AND SUPPORT FOR PUBLICATION: If you would like help with English language editing, or other article preparation support, Wiley Editing Services offers expert help, including English Language Editing, as well as translation, manuscript formatting, and figure formatting at www.wileyauthors.com/eeo/preparation. You can also find resources for Preparing Your Article for general guidance about writing and preparing your manuscript at www.wileyauthors.com/eeo/prepresources.

RIGOUR AND REPRODUCIBILITY: We recommend authors consult The Journal's Rigour and Reproducibility requirements to ensure that their manuscript is suitable for publication: https://physoc.onlinelibrary.wiley.com/pb-assets/hub-assets/physoc/documents/TJP-Rigour-and-Reproducibility-Requirements.pdf

REVISION CHECKLIST:

Check that your Methods section conforms to journal policy: https://jp.msubmit.net/cgi-bin/main.plex?form_type=display_requirements#methods.

Check that data presented conforms to the statistics policy: https://jp.msubmit.net/cgi-bin/main.plex?form_type=display_requirements#statistics.

Upload a full Response to Referees file. To create your 'Response to Referees': copy all the reports, including any comments from the Senior and Reviewing Editors, into a Microsoft Word, or similar, file and respond to each point, using font or background colour to distinguish comments and responses and upload as the required file type.

Please upload two versions of your manuscript text: one with all relevant changes highlighted and one clean version with no changes tracked. The manuscript file should include all tables and figure legends, but each figure/graph should be uploaded as separate, high-resolution files.

You may also upload:

- 'Potential Cover Art' for consideration as the issue's cover image
- Appropriate Supporting Information (Video, audio or data set: see https://jp.msubmit.net/cgi-bin/main.plex?form_type=display_requirements#supp).

We look forward to receiving your revised submission.

If you have any queries, please reply to this email and we will be pleased to advise.

Yours sincerely,

Richard Carson
Senior Editor
The Journal of Physiology

REQUIRED ITEMS

- Author photo and profile. First or joint first authors are asked to provide a short biography (no more than 100 words for one author or 150 words in total for joint first authors) and a portrait photograph. These should be uploaded and clearly labelled together in a Word document with the revised version of the manuscript. See Information for Authors for further details.

- Please upload separate high-quality figure files via the submission form.

- Please ensure that any tables are editable and in Word format, and wherever possible, embedded in the article file itself.

- Papers must comply with the Statistics Policy: https://jp.msubmit.net/cgi-bin/main.plex?form_type=display_requirements#statistics.

In summary:

- If n {less than or equal to} 30, all data points must be plotted in the figure in a way that reveals their range and distribution. A bar graph with data points overlaid, a box and whisker plot or a violin plot (preferably with data points included) are acceptable formats.

- If n > 30, then the entire raw dataset must be made available either as supporting information, or hosted on a not-for-profit repository, e.g. FigShare, with access details provided in the manuscript.

- 'n' clearly defined (e.g. x cells from y slices in z animals) in the Methods. Authors should be mindful of pseudoreplication.

- All relevant 'n' values must be clearly stated in the main text, figures and tables.

- The most appropriate summary statistic (e.g. mean or median and standard deviation) must be used. Standard Error of the Mean (SEM) alone is not permitted.

- Exact p values must be stated. Authors must not use 'greater than' or 'less than'. Exact p values must be stated to three significant figures even when 'no statistical significance' is claimed.

- Please include an Abstract Figure file and an Abstract Figure legend. An appropriate figure legend, which should not exceed 150 words in length, should be included in the main manuscript file. The Abstract Figure is a piece of artwork designed to give readers an immediate understanding of the research and should summarise the main conclusions. If possible, the image should be easily 'readable' from left to right or top to bottom. It should show the physiological relevance

of the manuscript so readers can assess the importance and content of its findings. Abstract Figures should not merely recapitulate other figures in the manuscript. Please try to keep the diagram as simple as possible and without superfluous information that may distract from the main conclusion(s). Abstract Figures must be provided by authors no later than the revised manuscript stage and should be uploaded as a separate file during online submission labelled as File Type 'Abstract Figure'. Please also ensure that you include the figure legend in the main article file. All Abstract Figures should be created using BioRender. Authors should use The Journal's premium BioRender account to export high-resolution images. Details on how to use and access the premium account are included as part of this email.

EDITOR COMMENTS

Reviewing Editor:
This manuscript examines the contribution of persistent inward currents to firing of phrenic and intercostal motoneurons during breathing. The reviewers appreciate the new insights provided in the manuscript and have raised a number of relatively minor concerns to be addressed in the final version.

REFEREE COMMENTS

Referee #1:

General Comments:

This is a very interesting and well-performed study estimating the contribution of persistent inward currents (PICs) to the discharge of motor units in the diaphragm muscle and parasternal intercostal motor units in humans. There is an extensive literature regarding the contribution of PICs in motoneurons innervating limb muscles in animal and human studies; however, the contribution of PICs in phrenic and intercostal motoneurons has not been clearly established. The investigators utilized a well-accepted technique of motor unit discharge rate hysteresis (delta F) to estimate the contribution of PICs. Using this technique, the investigators clearly demonstrated PICs in diaphragm motor unit discharge during quiet breathing. By contrast, discharge hysteresis (PICs) was not consistently observed in motor units of the 1st, 3rd and 5th parasternal intercostal muscles.

A minor criticism is the comparison between involuntary (quiet) and voluntary breathing. It is unclear what the investigators are exploring by this comparison. It is likely that the synaptic input to phrenic and parasternal intercostals differs between involuntary (brainstem) and voluntary (cerebrospinal) conditions. Are PICs a property of motoneurons or synaptic input? Did the investigators expect a difference? What novel information is gained by this comparison?

Another minor criticism is that the functional implications of differences in the contribution of PICs to prolonging discharge (beyond inspiratory airflow - i.e., post-inspiratory) in diaphragm motor units but not in parasternal intercostal motor units was not adequately discussed. Post-inspiratory activity of the diaphragm serves to brake expiratory airflow and prolong the expiratory phase (slower respiratory rate). The functional contribution of parasternal intercostal activation to airflow (inspiratory and post-inspiratory/expiratory) is relatively minor. With respect to phrenic motoneurons, a recent study in awake rats (Khurram et al. J Neurophysiol 2024) reported a marked decrease in the duration of post-inspiratory diaphragm activity during hypercapnia. Since hypercapnia increases inspiratory drive to phrenic motoneurons does the shortening of post-inspiratory activity reflect active inhibition of PICs?

Referee #2:

The purpose of this innovative study was to assess if persistent inward currents (PICs) contribute to control of motor unit activity in human inspiratory muscles. Previous research has demonstrated motor unit activity patterns during respiration which could indicate neuromodulation by PICs; however, the potential contribution of this mechanism has not been measured. The current study aimed to address this mechanistic question with motor unit pair analysis and firing duration ratio analysis on four inspiratory muscles. The authors present compelling and novel evidence that PICs contribute to prolonged motor unit firing in the diaphragm during automatic breathing. Additionally, variable results within the parasternal intercostal muscles during automatic and voluntary breathing suggest a need for further studies to address the neuromodulatory control of inspiratory muscles. This study fits well within the scope of the Journal of Physiology and would

be appealing to neurophysiologists and clinicians. Understanding how inspiratory muscles are controlled can eventually inform the improvement of ventilatory devices, respiratory therapies, medications, etc., and thus is vital to clinicians and researchers in many fields.

Minor concerns related to the introduction, results, and discussion are detailed below. Additionally, the authors need to address the limitations of their sample. Specifically there is only one female and the age difference between experiment one and two.

Introduction:

• Second paragraph: please be more clear which citations involve animal models. Some points made include citations of studies involving rats, cats, and humans, but it is not clear which statements apply to which models. The paragraph is framed by the mention of animal models, but there are human models included.

• Please provide information on the clinical or real-world significance, i.e., why do we care about the neural control of breathing?

Methods:

• I strongly recommend removing the female participant's data and reframing the article/title to state "in men," as applying this conclusion to all humans may be oversimplifying.

• Please address potential limitations in the interpretation of the results related to the age difference between groups. What was the rationale for using the young participants instead of middle-aged from Nguyen et al 2019? Would the middle-aged group not match experiment two better?

• Line 224: Please provide a figure/example of the visual feedback.

• Lines 245-246: Please provide more explanation of what qualifies adding or excluding.

Results:

• Line 371: Please remove "no evidence of" and state "there were no significant differences detected."

• Lines 407-419: Please clarify the rationale for presenting both the duration ratio and the percentage. Are they not stating the same thing?

o Please include a figure demonstrating the duration ratio differences discussed in lines 407-419. This could include the proportion of MUs with duration ratios <1 for each muscle and the EMM for the duration ratio.

• Line 420: Is this still discussing the estimated marginal mean of the duration ratio? The transition to table 2 in this paragraph seems out of place.

• Lines 437-440 are not written clearly. Additionally, lines 437-443 are already addressed in methods lines 333-342. Consider shortening the explanation of the unmatched analysis here especially since the matched and unmatched results showed no difference.

• Lines 475-482: Again, consider keeping only one of these metrics (duration ratio or percent of firing time in inspiration).

Discussion and Conclusions:

• Please consider condensing information to shorten.

• Lines 516-562: For clarity please consider breaking this paragraph down.

• Lines 556-562: For clarity, please reduce run on sentences and shorten language on the Denton model.

• Lines 579-595: Consider removing this or moving a shorter version to Directions for Future Research.

• Lines 631-674: Discussion of length of breath, functional relevance of short breaths, then future directions with intentionally long breaths could be shortened to one paragraph.

• Please discuss the limitations of the sample (small size, age, sex).

• Please include real-world importance of the findings .

END OF COMMENTS

Dear editors and reviewers,

Thank you for taking the time to review our manuscript and providing us with feedback. We have considered your feedback and made changes accordingly and believe the manuscript has been improved as a result. Please find below point-by-point responses to each question and description of how changes were made to the manuscript, which we hope you will find satisfactory. Changes are highlighted in blue (additions) or ~~red~~ (deletions). In this document, sometimes deletions were omitted for clarity, but both additions and deletions are highlighted in the revised manuscript. In the responses, the page numbers refer to the manuscript version with highlighted changes.

EDITOR COMMENTS

Reviewing Editor:

This manuscript examines the contribution of persistent inward currents to firing of phrenic and intercostal motoneourons during breathing. The reviewers appreciate the new insights provided in the manuscript and have raised a number of relatively minor concerns to be addressed in the final version.

Response

We thank you for the opportunity to revise the manuscript. We appreciated the feedback from the two experts and their recognition of the significance of our study. We have carefully addressed all the comments made by both reviewers. Please note that authors' affiliations have been updated.

REFEREE COMMENTS

Referee #1:

General Comments:

This is a very interesting and well-performed study estimating the contribution of persistent inward currents (PICs) to the discharge of motor units in the diaphragm muscle

and parasternal intercostal motor units in humans. There is an extensive literature regarding the contribution of PICs in motoneurons innervating limb muscles in animal and human studies; however, the contribution of PICs in phrenic and intercostal motoneurons has not been clearly established. The investigators utilized a well-accepted technique of motor unit discharge rate hysteresis (delta F) to estimate the contribution of PICs. Using this technique, the investigators clearly demonstrated PICs in diaphragm motor unit discharge during quiet breathing. By contrast, discharge hysteresis (PICs) was not consistently observed in motor units of the 1st, 3rd and 5th parasternal intercostal muscles.

Response:

We would like to thank the reviewer for taking the time to review the manuscript. We agree that, although an extensive body of literature has examined the contribution of PICs in motoneurons innervating limb muscles in both animal and human studies, their role in respiratory motoneurons has not yet been explored in humans, and this manuscript helps address this gap by providing new evidence and insight into the contribution of PICs within phrenic and parasternal intercostal motoneuron pools. We hope that this article will encourage research groups to pursue this important line of investigation in humans. You can find below your point-by-point responses. Page numbers are from the version with changes highlighted.

A minor criticism is the comparison between involuntary (quiet) and voluntary breathing. It is unclear what the investigators are exploring by this comparison. It is likely that the synaptic input to phrenic and parasternal intercostals differs between involuntary (brainstem) and voluntary (cerebrospinal) conditions. Are PICs a property of motoneurons or synaptic input? Did the investigators expect a difference? What novel information is gained by this comparison?

Response:

Prior to conducting this study, it was not possible to confidently predict the direction of any potential difference in PIC contribution during matched voluntary

breathing. This difficulty in expecting a difference in one or the other direction has now been made even more clear in our introduction (Page 6, Lines 17-18):

> ***In addition, it is difficult to postulate whether the magnitude of the contribution of PICs to inspiratory motor unit activity varies depending on whether these skeletal respiratory muscles are activated via bulbospinal (automatic) pathways, or corticospinal (voluntary pathways). On one hand, greater serotonin release could occur during voluntary motor activity (Veasey et al., 1995; Jacobs et al., 2002) and enhance PIC activity (Hounsgaard et al., 1988). Conversely, activation of inhibitory spinal interneurons during both voluntary inspiration and expiration may lead to a concomitant increase in inhibition of inspiratory motoneurons (Andersen & Sears, 1970; Merrill & Fedorko, 1984). Since PICs in limb motoneurons are highly sensitive to inhibitory inputs (Heckman et al., 2008; Orssatto et al., 2022; Mesquita et al., 2022, 2024), this could attenuate their contribution.***

Regarding one of the reviewer's question, PICs are indeed an intrinsic property of motoneurons, as they are induced by voltage-gated calcium and sodium channels. The activity of these channels is the primary contributor in transforming these cells from linear transducers of synaptic input into highly non-linear actuators. However, it is important to note that the magnitude of the activity of these channels is strongly modulated by prior neural discharge (i.e., excitatory input and especially inhibitory input) and the neuromodulatory state (i.e., serotonergic and noradrenergic input). Thus, while PICs are an intrinsic property of motoneurons, their contribution to motor output depends largely on the balance of excitatory, inhibitory, and monoaminergic inputs.

The comparison between quiet and voluntary breathing was therefore intended to test whether altering the source and organisation of descending drive (bulbospinal vs. corticospinal), while matching the mechanical output, would measurably alter the effective contribution of PICs (estimated via ΔF). Although the ventilatory output was matched, the underlying synaptic and neuromodulatory conditions could plausibly differ and thereby modulate PIC expression.

Our findings indicate that ΔF was not significant or consistent in parasternal motor units during quiet breathing, and it did not differ during voluntary matched breaths. These results suggest that PICs do not make a substantial functional contribution to parasternal motoneuron firing, and that changing the source and organisation of the descending drive strategy does not unmask such a contribution.

We have expanded the Discussion (Page 24, Lines 1-16) to more explicitly interpret the absence of significant ΔF in both conditions. We thank the reviewer for this input as we believe that this additional paragraph has strengthened our discussion.

> ***In the present study, we observed no significant differences in ΔF between quiet and voluntary breaths. This indicates that altering the source of descending drive to inspiratory motor units (i.e., bulbospinal vs corticospinal), while matching voluntary output, did not measurably alter the estimated contribution of PICs to motoneuron firing. Although an increase in voluntary drive could be associated with changes in monoaminergic input (Veasey et al., 1995; Jacobs et al., 2002) or spinal inhibitory activity (Andersen & Sears, 1970; Merrill & Fedorko, 1984) – both of which are known to modulate PIC expression (Hounsgaard et al., 1988; Heckman et al., 2008; Orssatto et al., 2022; Mesquita et al., 2022, 2024) – these mechanisms were not directly assessed in the present study. Therefore, the absence of differences in ΔF may reflect that these potential modulatory influences associated with voluntary drive were minimal. Alternatively, voluntary breathing may have had a concurrent facilitatory and inhibitory effect on PICs, resulting in no net change in firing prolongation. Finally, given the low and inconsistent magnitude of the estimated PIC-associated firing prolongation in this parasternal intercostal motor units, another possibility is that a floor effect may have constrained the detectable range of PIC modulation.***

Another minor criticism is that the functional implications of differences in the contribution of PICs to prolonging discharge (beyond inspiratory airflow - i.e., post-inspiratory) in diaphragm motor units but not in parasternal intercostal motor units was not adequately discussed. Post-inspiratory activity of the diaphragm serves to brake expiratory airflow and prolong the expiratory phase (slower respiratory rate). The

functional contribution of parasternal intercostal activation to airflow (inspiratory and post-inspiratory/expiratory) is relatively minor. With respect to phrenic motoneurons, a recent study in awake rats (Khurram et al. J Neurophysiol 2024) reported a marked decrease in the duration of post-inspiratory diaphragm activity during hypercapnia. Since hypercapnia increases inspiratory drive to phrenic motoneurons does the shortening of post-inspiratory activity reflect active inhibition of PICs?

Response:

We have now expanded the discussion on post-inspiratory activity in accordance to the reviewer's input. The potential role of PICs in hypercapnia-induced modulation of post-inspiratory activity is highlighted as an interesting avenue for future investigation (Pages 27-28, From line 12):

> ***Interestingly, the proportion of motor units that continued to fire during expiration (i.e., duration ratio < 1) was higher in diaphragmatic motor units than in the 1st and 3rd parasternal intercostals. As an interesting supplementary finding, the computation of duration ratios for parasternal intercostal motor units in this study further underscores their rostro-caudal gradient with differences in the timing and firing rates of motor unit activity during voluntary breathing, as previously reported (Hudson et al., 2011b). Future studies in animal models utilising more precise quantification of PIC magnitudes could investigate the impact of blocking or enhancing PICs on the duration of post-inspiratory activity and whether such an effect would have a functional relevance such as modulating expiratory airflow which could affect lung volumes and respiratory mechanics (O'Donnell & Laveneziana, 2006). In animal models, increased expiratory muscle activity has been observed during hypercapnia (Abdala et al., 2009; Molkov et al., 2010; Flor et al., 2020), a condition in which efficient CO_2 elimination becomes crucial. Functionally, prolonged post-inspiratory activity under such conditions could be counterproductive to the goal of augmenting expiratory airflow. Consistent with this notion, a marked decrease in the duration of post-inspiratory diaphragm activity was recently reported in awake rats (Khurram et al., 2024). If PICs contribute to post-inspiratory activity, one could postulate that activation of inhibitory spinal interneurons during states of enhanced***

expiratory drive (Andersen & Sears, 1970; Merril & Fedorko, 1984) may suppress PIC-mediated inspiratory activity, thereby shortening its persistence into expiration. Conversely, intermittent hypercapnia could have the opposite effect, given its possible facilitatory effect in human inspiratory motoneuron pools (Welch et al., 2022; Mesquita, 2022a). Futures studies could usefully explore whether such state-dependent modulations of post-inspiratory activity are also present in humans and, if so, whether they are mediated by PICs.

References:

Abdala, APL, Rybak, IA, Smith, JC, & Paton, JFR (2009). Abdominal expiratory activity in the rat brainstem-spinal cord in situ: Patterns, origins and implications for respiratory rhythm generation. J Physiol, 587(14), 3539–3559.

Andersen P & Sears TA (1970). Medullary activation of intercostal fusimotor and alpha motoneurones. J Physiol 209, 739–755.

Flor, KC, Barnett, WH, Karlen-Amarante, M, Molkov, YI, & Zoccal, DB (2020). Inhibitory control of active expiration by the Bötzinger complex in rats. Journal of Physiology, 598(21), 4969–4994.

Heckman CJ, Hyngstrom AS & Johnson MD (2008). Active properties of motoneurone dendrites: Diffuse descending neuromodulation, focused local inhibition. J Physiol 586, 1225–1231.

Hounsgaard J, Hultborn H, Jespersen B & Kiehn O (1988). Bistability of alpha-motoneurones in the decerebrate cat and in the acute spinal cat after intravenous 5-hydroxytryptophan. J Physiol 405, 345–367.

Hudson AL, Gandevia SC & Butler JE (2011b). Common rostrocaudal gradient of output from human intercostal motoneurones during voluntary and automatic breathing. Respir Physiol Neurobiol 175, 20–28.

Jacobs BL, Martín-Cora FJ & Fornal CA (2002). Activity of medullary serotonergic neurons in freely moving animals. Brain Res Rev 40, 45–52.

Khurram, OU, Kantor-Gerber, MJ, Mantilla, CB, & Sieck, GC (2024). Hypercapnia impacts neural drive and timing of diaphragm neuromotor control. J Neurophysiol 132(6), 1966–1976.

Merrill EG & Fedorko L (1984). Monosynaptic inhibition of phrenic motoneurons: A long descending projection from Botzinger neurons. Journal of Neuroscience 4, 2350–2353.

Mesquita, RNO (2022a). Concurrent exposure to (acute intermittent) hypoxia and hypercapnia: a promising therapeutic cocktail for neuroplasticity? The J Physiol, 600(13), 3017–3019.

Mesquita RNO, Taylor JL, Trajano GS, Škarabot J, Holobar A, Gonçalves BAM & Blazevich AJ (2022b). Effects of reciprocal inhibition and whole-body relaxation on persistent inward currents estimated by two different methods. J Physiol, 600, 2765–2787.

Mesquita RNO, Taylor JL, Heckman CJ, Trajano GS & Blazevich AJ (2024). Persistent inward currents in human motoneurons: Emerging evidence and future directions. J Neurophysiol 132, 1278–1301.

Molkov, YI, Abdala, APL, Bacak, BJ, Smith, JC, Paton, JFR, & Rybak, IA (2010). Late-Expiratory Activity: Emergence and Interactions With the Respiratory CPG. Journal of Neurophysiology, 104(5), 2713.

O'Donnell DE & Laveneziana P (2006). The clinical importance of dynamic lung hyperinflation in COPD. COPD 3, 219–232.

Orssatto LBR, Fernandes GL, Blazevich AJ & Trajano GS (2022). Facilitation–inhibition control of motor neuronal persistent inward currents in young and older adults. J Physiol 600, 5101–5117.

Veasey SC, Fornal CA, Metzler CW & Jacobs BL (1995). Response of serotonergic caudal raphe neurons in relation to specific motor activities in freely moving cats. Journal of Neuroscience 15, 5346–5359.

Welch, JF, Nair, J, Argento, PJ, Mitchell, GS, & Fox, EJ (2022). Acute intermittent hypercapnic-hypoxia elicits central neural respiratory motor plasticity in humans. J Physiol 10, 2515–2533.

Referee #2:

The purpose of this innovative study was to assess if persistent inward currents (PICs) contribute to control of motor unit activity in human inspiratory muscles. Previous research has demonstrated motor unit activity patterns during respiration which could indicate neuromodulation by PICs; however, the potential contribution of this mechanism has not been measured. The current study aimed to address this mechanistic question with motor unit pair analysis and firing duration ratio analysis on four inspiratory muscles. The authors present compelling and novel evidence that PICs contribute to prolonged motor unit firing in the diaphragm during automatic breathing. Additionally, variable results within the parasternal intercostal muscles during automatic and voluntary breathing suggest a need for further studies to address the neuromodulatory control of inspiratory muscles. This study fits well within the scope of the Journal of Physiology and would be appealing to neurophysiologists and clinicians. Understanding how inspiratory muscles are controlled can eventually inform the

improvement of ventilatory devices, respiratory therapies, medications, etc., and thus is vital to clinicians and researchers in many fields.

Minor concerns related to the introduction, results, and discussion are detailed below. Additionally, the authors need to address the limitations of their sample. Specifically there is only one female and the age difference between experiment one and two.

Response:

We would like to thank the reviewer for taking the time to review the manuscript. We agree that our data advance the knowledge on neuromotor control of human inspiratory motoneurons which is the foundation for developing therapeutic approaches for functional recovery of ventilatory and non-ventilatory behaviours. We have highlighted this clinical implication and addressed the other minor concerns, as detailed below.

Introduction:

• Second paragraph: please be more clear which citations involve animal models. Some points made include citations of studies involving rats, cats, and humans, but it is not clear which statements apply to which models. The paragraph is framed by the mention of animal models, but there are human models included.

Response:

We have now made this information more explicit in the paragraph. In addition, to improve conceptual continuity (given that the paragraph ends with the rationale for estimating PICs in human inspiratory motor units) we have replaced some references describing PIC-induced effects on motoneuron output with corresponding evidence from human motor unit studies. We believe these revisions clarify the translational progression from animal findings to human investigations and improve the overall coherence of the paragraph (Page 4, Lines 25-30).

> ***Yet, this is just one possible aspect of the multifaceted effects of PICs and other intrinsic properties on human motoneuron output (Binder et al., 2020; Chardon et al., 2023; Mesquita et al., 2024). Other non-linear firing patterns***

have been widely described in human motor unit recordings: firing hysteresis (Gorassini et al., 2002a), firing saturation (Fuglevand et al., 2015), warm-up (Gorassini et al., 2002b), and self-sustained firing (Gorassini et al., 1998).

Please provide information on the clinical or real-world significance, i.e., why do we care about the neural control of breathing?

<u>Response:</u>

The following sentence has been added to the end of the Introduction (Page 7, Lines 2-6):

Establishing whether PICs contribute to inspiratory motoneuron firing is essential for a mechanistic understanding of the neural control of respiratory motoneurons. Such knowledge would inform whether interventions known to modulate PIC activity in human spinal motoneurons are likely to be effective for targeting motor output during ventilatory and non-ventilatory behaviours.

Methods:

• I strongly recommend removing the female participant's data and reframing the article/title to state "in men," as applying this conclusion to all humans may be oversimplifying.

<u>Response:</u>

The study was not designed or powered to examine sex differences, and there was no *a priori* hypothesis regarding sex-related effects. We therefore consider that it would not be beneficial to remove a participant *post hoc* based solely on sex, particularly given the small sample size in these challenging human experiments and the absence of a strong physiological rationale for such exclusion. We have retained the data as collected and now explicitly acknowledge in the second paragraph of "Directions for future research" that the cohort was predominantly male and that future studies with larger, sex-balanced samples are needed to

determine whether sex differences in PIC contribution to respiratory motoneuron firing exist.

> ***While the current study provides novel insights into the contribution of PICs to inspiratory motoneuron firing in humans, future research is needed to corroborate or refine our findings. For example, interventions that elicit inhibitory inputs onto motoneurons (Orssatto et al., 2022; Mesquita et al., 2022b; Pearcey et al., 2022; Gomes et al., 2024), with ingestion of monoaminergic drugs (Udina et al., 2010; D'Amico et al., 2013; Goodlich et al., 2023; Mohammadalinejad et al., 2024), or studies examining ageing (Hassan et al., 2021; Orssatto et al., 2021a; Mohammadalinejad et al., 2024; Guo et al., 2024) have consistently demonstrated modulation of PIC activity. If differences in estimates of PIC activity in inspiratory motoneurons are observed in future studies employing similar experimental approaches, it would further support the hypothesis that PICs can play an important modulatory role in respiratory motoneuron output. Moreover, given the predominantly male sample and limited cohort size, future studies with larger and sex-balanced samples are required to confirm these findings and to determine whether sex-related differences in PIC contribution to respiratory motoneuron firing are present. While the only studies that have formally examined sex differences in limb motoneuron pools reported greater PIC estimates in females in most (Jenz et al., 2023; Lecce et al., 2025; Yacyshyn et al., 2025) but not all cases (Mesquita et al., 2022b, 2023), it remains unknown whether sex differences occur in respiratory motoneurons.***

• Please address potential limitations in the interpretation of the results related to the age difference between groups. What was the rationale for using the young participants instead of middle-aged from Nguyen et al 2019? Would the middle-aged group not match experiment two better?

Response:

The younger cohort from Nguyen et al. (2019) was selected because a substantial proportion of diaphragm motor unit action potentials in the middle-aged group were multiphasic. The reviewer does address a valid point and we acknowledge that the age difference between groups represents a potential limitation when interpreting the results and something to be considered in future work. We have now explicitly addressed this in the Discussion (Page 23, Lines 25-31):

> ***Finally, we cannot rule out the possibility that the variability and potential absence of significant PIC contribution in parasternal intercostal motoneurons may be influenced by age rather than muscle-specific factors, given the older age of the parasternal intercostal cohort (30-58 years) compared to the diaphragm cohort (26-26 years). However, this possibility is not supported by recent evidence (Mohammadalinejad et al., 2024) showing no significant differences in ΔF tibialis anterior motor units between young (18-28 years) and middle-aged adults (32-53 years).***

• Line 224: Please provide a figure/example of the visual feedback.

<u>Response:</u>

Readers are now directed to Figure 1 of Hudson et al., (2011b), which provides a detailed illustration of the experimental setup and visual feedback procedure for parasternal intercostal motor unit recordings (Page 9, Line 5).

> ***In Experiment 2, an initial 30-s period of quiet, automatic breathing was recorded. During this period, participants did not receive visual feedback of lung volume, were not instructed on their breathing and were unaware that recordings were taking place. During the quiet breathing period, a researcher established a target lung volume for the voluntary breaths. The target increased through inspiration and was designed to match the mean inspiratory flow and tidal volume of the participant's quiet breathing. With the electrode in the same position, participants were provided with visual feedback of inspiratory lung volume and instructed to closely follow the target. There was no feedback of expiratory lung volume (see Figure 1 in Hudson et al. (2011b)). Participants typically needed six attempts (range: 3-20) to achieve***

three successful voluntary breaths that closely matched the target (as judged by a dedicataed investigator). Once this was completed, the electrode was repositioned to a new recording site. The procedure was similar for each interspace, and EMG recordings were sampled from 6-10 sites in each interspace.

Figure 1 legend (Hudson et al., 2011b) provides further methodological details. Panels also show quiet and voluntary breaths, including inspiratory lung volume traces, highlighting the target and tracking performance.

• Lines 245-246: Please provide more explanation of what qualifies adding or excluding.

<u>Response:</u>

We have clarified this point in the revised manuscript. All motor unit activity underwent visual inspection to verify the automatic detection. Motor unit firings were added or excluded where necessary to correct for false positives or missed discharges (e.g. when two motor units fired simultaneously, the resulting change in motor unit action potential shape could decrease the ability for the automatic detection algorithm to identify a true firing), based on simultaneous comparison of the raw EMG, motor unit templates, and instantaneous discharge frequency plots (Page 9, Lines 25-29).

For each recording site, three sequential quiet breaths (Experiments 1 and 2) and three voluntary breaths that were well-matched to the target of inspiratory lung volume (Experiment 2) were initially chosen for offline analysis. Individual motor unit activity was identified in these breaths in a commercial software package (Spike 2, Cambridge Electronic Design, UK), as previously described (Gandevia et al., 1996). Briefly, trigger levels were manually set to identify spikes of an appropriate signal-to-noise ratio. Then, EMG data were sorted into motor unit templates based on the shape of their action potentials, which could be tracked between breaths. A customised script enabled the visual inspection of each motor unit template. All motor unit activity was visually inspected to verify the automatic detection. **Motor unit**

> ***firings could be added or excluded as necessary to correct for false positives or missed discharges based on comparison of the raw EMG, motor unit templates, and instantaneous discharge frequency plots viewed simultaneously.*** ~~***, and the firing frequency pattern could be viewed for each motor unit.***~~

Results:

• Line 371: Please remove "no evidence of" and state "there were no significant differences detected."

<u>Response:</u>

This has been amended as requested (Page 14, Line 3).

> ~~***There was no evidence of significant differences***~~ ***Significant differences were not detected ($F_{(23,3)}$ = 0.91, p = 0.449) between muscles on ΔF scores, and the estimated ΔF mean scores and 95% confidence intervals were as follows: diaphragm: 2.29 Hz [1.08, 3.5]; 1st parasternal intercostal: 1.78 Hz [0.43, 3.12]; 3rd: 1.08 Hz [-0.39, 2.55]; 5th: 1.55 Hz [-0.04, 3.13; Figure 2).***

• Lines 407-419: Please clarify the rationale for presenting both the duration ratio and the percentage. Are they not stating the same thing?

<u>Response:</u>

Although the duration ration and the proportion of firing time during inspiration are mathematically related, we chose to present both for complementary reasons. The inspiration-to-expiration duration ratio (-1 to 1) has been reported in previous studies (Afsharipour et al., 2020; Hassan et al., 2021; Jenz et al., 2023; Mohammadalinejad et al., 2024), and we included it to facilitate comparison with existing literature and to provide a symmetric index of inspiratory-expiratory motor unit activity. However, as this metric may be less intuitive to interpret, we additionally expressed motor unit activity as the proportion of total firing time occurring during inspiration (0-100%). We have clarified this rationale in the Methods section (Page 11, Lines 15-21):

An inspiration-to-expiration motor unit firing duration ratio was also calculated to assess asymmetry in motor unit firing during the inspiratory and expiratory phases, which were identified from lung volume (using a technique adapted from the "self sustained firing duration" reported in Afsharipour et al. (2020)). The duration ratio was determined by subtracting the duration of motor unit firing during expiration from the duration of motor unit firing during inspiration, and then dividing the difference by the total duration of motor unit firing. Duration ratios were averaged across breaths. The resulting values ranged from -1 to 1, with a value of 1 indicating motor units active only during inspiration. A value of -1 would have corresponded to exclusive activity during expiration. This symmetric index has been used in previous studies (Afsharipour et al., 2020; Hassan et al., 2021; Jenz et al., 2023; Mohammadalinejad et al., 2024) and allows direct comparison with existing literature. To provide a more intuitive measure, we also expressed motor unit activity as the proportion (percentage) of total firing time occurring during inspiration. Although mathematically related, the two metrics emphasise comparability with prior work and interpretability, respectively.

o Please include a figure demonstrating the duration ratio differences discussed in lines 407-419. This could include the proportion of MUs with duration ratios <1 for each muscle and the EMM for the duration ratio.

<u>**Response:**</u>

The duration ratio analysis was included as a secondary descriptive measure and is not central to the primary aims of the study. These and the proportion of motor units with duration ratios < 1 are already clearly reported in the text. Therefore, we have not added an additional figure, as this would be unlikely to substantially enhance interpretation and may detract from the focus on the primary outcome (ΔF). Unlike the duration ratio, ΔF is a mechanistically grounded and experimentally validated estimate of PIC contribution to firing hysteresis, supported by intracellular recordings in animal models and computational simulations, as outlined in the Introduction. For this reason, we prioritised clear presentation of ΔF as the main outcome variable.

• Line 420: Is this still discussing the estimated marginal mean of the duration ratio? The transition to table 2 in this paragraph seems out of place.

Response:

We have revised the paragraph to clarify that the initial section refers specifically to the estimated marginal means of the duration ratios, and we now more clearly signal the transition to other secondary variables presented in Table 2 (Page 17, Lines 4-11).

> ***A significant effect of muscle was observed (F(21,3) = 7.36, p = 0.001), with post-hoc comparisons revealing that the 5th parasternal intercostal muscle exhibited significantly lower duration ratios compared to both the 1st (p = 0.003) and the 3rd (p < 0.001) parasternal intercostal muscles. No other statistically significant differences were detected by the linear mixed model, despite ~~a~~ the lower absolute estimated marginal mean value for diaphragm MU duration ratios~~, the linear mixed model analysis did not reveal any other statistically significant differences among the muscles~~. Estimated marginal means and confidence intervals for other secondary variables (i.e., peak, averaged and ranges of smoothed firing rates, and ~~for~~ recruitment thresholds), as well as indication of significant differences between muscles can also be found in Table 2.***

• Lines 437-440 are not written clearly. Additionally, lines 437-443 are already addressed in methods lines 333-342. Consider shortening the explanation of the unmatched analysis here especially since the matched and unmatched results showed no difference.

Response:

The reviewer is right that this was already described to a certain extent in the methods section. Thus, the results section was now shortened and a few details added in the methods section.

Methods (Page 12, Lines 26-27):

> ***Tracking the same pairs between quiet and voluntary conditions did not permit a thorough examination of potential differences in ΔF for the 3rd and 5th parasternal intercostal muscles. Specifically, only 6 test units (from three participants) were identified for the 3rd, and 2 test units (from two participants) identified for the 5th interspaces. Thus, an additional analysis was conducted (Variable ~ muscle + (1 | Participant)), considering all test units identified in quiet and voluntary conditions (i.e., tracking of pairs or test units not contemplated). This approach enabled an examination of potential differences in the 3rd parasternal intercostal muscle but remained limited for the 5th parasternal intercostal muscle, with only 3 test units identified during voluntary breathing.***

Results (Page 17-18, From line 25):

> ***~~Potential differences in ΔF while tracking the same pairs between conditions could not be assessed for the 3rd and 5th parasternal intercostal muscles, as only six test units (from three participants) and two test units (from two participants), respectively, could be tracked. To address this limitation, an additional unmatched motor unit analysis was conducted, in which tracking of the same test units was not considered in the model, but only including participants with motor units identified in both conditions.~~ The unmatched analysis (i.e., tracking of the same test units was not considered in the model) allowed for the comparison of ΔF scores between quiet and voluntary breathing from 25 and 18 test units (n = 4) for the 1st parasternal intercostal (Figure 3, central panel) and from 19 and 15 test units (n = 3) for the 3rd parasternal intercostal muscle (Figure 3, right panel).***

• Lines 475-482: Again, consider keeping only one of these metrics (duration ratio or percent of firing time in inspiration).

Response:

This has been previously addressed.

Discussion and Conclusions:

• Please consider condensing information to shorten.

Response:

We have revised the Discussion to improve conciseness in the sections highlighted by Reviewer 2. At the same time, we have incorporated the additional points requested by Reviewer 1. We have aimed to balance brevity with sufficient detail to address both reviewers' comments.

• Lines 516-562: For clarity please consider breaking this paragraph down.

Response:

In this paragraph we clarify potential differences between our findings in human diaphragmatic motor units and a prior animal study (Enríquez Denton et al. (2012)). We remind the reader that there are distinct non-linear behaviours of motoneurons that could be induced by PICs (e.g., firing prolongation in our study versus synaptic input amplification in Enríquez Denton et al. (2012)) and explain why diaphragm ΔF scores in our study may reflect PIC contributions even if one prior cat study suggested minimal PIC involvement. Additionally, we highlight methodological, species, and motoneuron population differences that could influence PIC detection, as well as other intrinsic properties that could "contaminate" ΔF interpretations, ensuring readers interpret our results in the appropriate physiological and experimental context.

Some changes were made for conciseness (Pages 20-21, From Line 25):

The observation of ΔF scores in diaphragmatic motor units with a magnitude indicative of PIC-induced firing prolongation (i.e., mean 2.29 Hz) contrasts with the suggested absence of PIC involvement in cat phrenic inspiratory motoneurons (Enríquez Denton et al., 2012). However, there are several methodological differences between the studies. First, Enriquez Denton and colleagues (2012) assessed voltage-dependent amplification of synaptic inputs (i.e., the increase in peak depolarisation amplitude during inspiration at more depolarised membrane potentials) whereas the current study assessed recruitment-derecruitment hysteresis (ΔF in human studies or ΔI, change in injected current, for cellular studies). In motoneuron firing, synaptic input amplification is expected to lead to the non-linear behaviours of firing acceleration on recruitment and saturation (Heckman et al., 2008; Powers & Heckman, 2017; Binder et al., 2020; Zero et al., 2022; Beauchamp et al., 2023; Chardon et al., 2023; Mesquita et al., 2024), whereas firing prolongation is believed to have a different underlying biophysical mechanism (Shapiro & Lee, 2007; Binder et al., 2020). Thus, PICs could potentially contribute to firing prolongation in diaphragmatic motoneurons without necessarily affecting synaptic input amplification. Second, the electrodes used in Enríquez Denton et al. (2012) contained the local anaesthetic derivative QX-314, which could have blocked PIC activity (Lee & Heckman, 1999), potentially limiting detection of PIC involvement. Third, the present study was conducted in humans as opposed to cats, raising the possibility that the existence and contribution of PIC channels to inspiratory motoneuron firing may differ between species. Fourth, different populations of diaphragmatic motoneurons were likely examined in the two studies. While it remains unclear whether PIC magnitude varies with motoneuron size (Mesquita et al., 2024), the present study likely identified smaller, lower-threshold diaphragmatic motor units during quiet breathing (Sieck & Fournier, 1989; Seven et al., 2013). In contrast, the sample of phrenic motoneurons identified in Enríquez Denton et al. (2012) was biased towards larger motoneurons. Finally, it cannot be ruled out that mechanisms independent of PICs could have contributed to positive ΔF scores, including slow inactivation of Kv 1.2 channels in the axon initial segment (Bos et al.,

2018) and spike frequency adaptation (Revill & Fuglevand, 2011). Although NMDA receptor activity has not been directly linked to recruitment-derecruitment hysteresis, the proposed role of NMDA-mediated currents in synaptic input amplification in cat phrenic motoneurons (Enríquez Denton et al. (2012)), and the capacity of NMDA to induce membrane potential oscillations that drive rhythmic motoneuron firing in adult rodents (Manuel et al., 2012), suggests that a potential contribution of NMDA receptors to the ΔF scores observed here cannot be entirely excluded.

• Lines 556-562: For clarity, please reduce run on sentences and shorten language on the Denton model.

Response:

Addressed in the previous comment.

• Lines 579-595: Consider removing this or moving a shorter version to Directions for Future Research.

Response:

A shorter version has been moved to Directions for Future Research (Pages 28-29, from line 23):

Could PICs contribute to firing prolongation in parasternal intercostal muscles in other physiological contexts, such as~~? One possible example is~~ during trunk rotations? Unlike the diaphragm (Hudson et al., 2011a), the parasternal intercostal muscles significantly contribute to ipsilateral rotations, and their phasic inspiratory activity is greatly increased ~~during~~ when breathing occurs during such~~in the presence of an ipsilateral~~ rotations (Hudson et al., 2010, 2011a, 2017). ~~An increase in the output of parasternal motoneurons is reflected in a higher number of motor units being recruited, as well as earlier and faster motor unit firing. It has been postulated that~~ This increased activity could be driven by greater descending excitatory inputs from the cortex or medulla, as well as afferent inputs (Hudson et al., 2010). As PIC channels are

voltage-gated (Svirskis & Hounsgaard, 1997; Li et al., 2004), ~~an~~ increased excitatory drive could augment PIC-induced non-linearities, as ~~directly~~ demonstrated in cat motoneurons (Hultborn et al., 2003) and inferred ~~during examination of~~ in human motor unit ~~firing patterns~~ recordings (Orssatto et al., 2021b; Goodlich et al., 2023; Mackay et al., 2023; Lapole et al., 2024). ~~Thus,~~ Future studies could ~~usefully~~ explore whether ~~there is a significant contribution of~~ PICs significantly contribute to parasternal motoneuron firing during non-respiratory ipsilateral rotations and during inspiratory activity in the presence of such trunk rotations.

• Lines 631-674: Discussion of length of breath, functional relevance of short breaths, then future directions with intentionally long breaths could be shortened to one paragraph.

<u>Response:</u>

We have intentionally kept the two-paragraph structure, as each addresses a distinct conceptual point. The first paragraph highlights the physiology of PICs (i.e., the relatively slow kinetics of PIC activation) and the constraints of the experimental context: the relatively short breath duration could mean that PICs were either not fully recruited during a single breath or remained partially active between breaths. This is important for understanding how the brevity of respiratory cycles may limit (or alternatively, sustain) the contribution of PICs to motoneuron firing.

The second paragraph addresses the methodological assumption that the lower-threshold "control" unit reflects the synaptic input to higher-threshold "test" units. In limb studies, pairs with recruitment intervals between the two units that are shorter than ~1 s are typically excluded to preserve this assumption, which was not possible here due to the short breath durations. Nevertheless, this limitation is likely mitigated by (i) the potential persistence of PICs between breaths addressed in the previous paragraph, (ii) the absence of significant correlations between recruitment intervals and ΔF scores, and (iii) the low likelihood that control units included marked secondary-range firing, which would have reduced rate-to-rate correlations.

Thus, the first paragraph contextualises PIC physiology and the constraints of the experimental conditions, while the second focuses on data analysis considerations and the implications of the experimental context for ΔF quantification. Future studies with longer-duration breaths could help reduce these confounds and explore additional metrics of PIC contribution, as then noted at the end of the second paragraph.

We considered it essential to provide this level of detail in this part of the Discussion, as it allows readers to fully appreciate the physiological and methodological nuances of ΔF estimation in respiratory motoneurons. This clarity is critical for readers to be able to evaluate the mechanistic insights we offer into intrinsic motoneuron excitability, in line with the journal's emphasis on advancing the mechanistic understanding of human and animal physiology.

- Please discuss the limitations of the sample (small size, age, sex).

<u>Response:</u>

This has been previously addressed.

- Please include real-world importance of the findings.

<u>Response:</u>

The following paragraph has been added to the end of the section Directions for Future Research (Page 29, Lines 23-28):

> ***Establishing whether PICs contribute to inspiratory motoneuron firing is an important step towards informing whether interventions known to modulate PIC activity in spinal motoneurons are likely to be effective for targeting respiratory motor output. The mechanistic knowledge presented here could also motivate future examinations of whether altered intrinsic motoneuron excitability plays a role in compromised respiratory motor output in pathological conditions.***

References:

Afsharipour B, Manzur N, Duchcherer J, Fenrich KF, Thompson CK, Negro F, Quinlan KA, Bennett DJ & Gorassini MA (2020). Estimation of self-sustained activity produced by persistent inward currents using firing rate profiles of multiple motor units in humans. J Neurophysiol 124, 63–85.

Beauchamp JA, Pearcey GEP, Khurram OU, Chardon M, Wang YC, Powers RK, Dewald JPA & Heckman CJ (2023). A geometric approach to quantifying the neuromodulatory effects of persistent inward currents on individual motor unit discharge patterns. J Neural Eng 20, 016034.

Bennett DJ, Li Y, Harvey PJ & Gorassini M (2001a). Evidence for plateau potentials in tail motoneurons of awake chronic spinal rats with spasticity. J Neurophysiol 86, 1972–1982.

Binder MD, Powers RK & Heckman CJ (2020). Nonlinear Input-Output Functions of Motoneurons. Physiology (Bethesda) 35, 31–39.

Bos R, Harris-Warrick RM, Brocard C, Demianenko LE, Manuel M, Zytnicki D, Korogod SM & Brocard F (2018). Kv1.2 Channels Promote Nonlinear Spiking Motoneurons for Powering Up Locomotion. Cell Rep 22, 3315–3327.

Chardon MK, Wang YC, Garcia M, Besler E, Beauchamp JA, D'Mello M, Powers RK, Heckman CJ & Preprint R (2023). Supercomputer framework for reverse engineering firing patterns of neuron populations to identify their synaptic inputs. elife 12, RP90624.

D'Amico JM, Murray KC, Li Y, Chan KM, Finlay MG, Bennett DJ & Gorassini MA (2013). Constitutively active 5-HT2/α1 receptors facilitate muscle spasms after human spinal cord injury. J Neurophysiol 109, 1473–1484.

Enríquez Denton M, Wienecke J, Zhang M, Hultborn H & Kirkwood PA (2012). Voltage-dependent amplification of synaptic inputs in respiratory motoneurones. J Physiol 590, 3067–3090.

Fuglevand AJ, Lester RA & Johns RK (2015). Distinguishing intrinsic from extrinsic factors underlying firing rate saturation in human motor units. J Neurophysiol 113, 1310–1322.

Gomes MM, Jenz ST, Beauchamp JA, Negro F, Heckman CJ & Pearcey GEP (2024). Voluntary co-contraction of ankle muscles alters motor unit discharge characteristics and reduces estimates of persistent inward currents. J Physiol 602, 4237–4250.

Goodlich BI, Del Vecchio A, Horan SA & Kavanagh JJ (2023). Blockade of 5-HT2 receptors suppresses motor unit firing and estimates of persistent inward currents during voluntary muscle contraction in humans. Journal of Physiology 601, 1121–1138.

Gorassini MA, Bennett DJ & Yang JF (1998a). Self-sustained firing of human motor units. Neurosci Lett 247, 13–16.

Gorassini M, Yang JF, Siu M & Bennett DJ (2002a). Intrinsic activation of human motoneurons: reduction of motor unit recruitment thresholds by repeated contractions. J Neurophysiol 87, 1859–1866.

Gorassini M, Yang JF, Siu M & Bennett DJ (2002b). Intrinsic activation of human motoneurons: Possible contribution to motor unit excitation. J Neurophysiol 87, 1850–1858.

Guo Y, Jones EJ, Škarabot J, Inns TB, Phillips BE, Atherton PJ & Piasecki M (2024). Common synaptic inputs and persistent inward currents of vastus lateralis motor units are reduced in older male adults. Geroscience 3, 1–13.

Hassan AS, Fajardo ME, Cummings M, McPherson LM, Negro F, Dewald JPA, Heckman CJ & Pearcey GEP (2021). Estimates of persistent inward currents are reduced in upper limb motor units of older adults. Journal of Physiology 599, 4865–4882.

Heckman CJ, Hyngstrom AS & Johnson MD (2008). Active properties of motoneurone dendrites: Diffuse descending neuromodulation, focused local inhibition. Journal of Physiology 586, 1225–1231.

Heckman CJ, Gorassini MA & Bennett DJ (2005). Persistent inward currents in motoneuron dendrites: Implications for motor output. Muscle Nerve 31, 135–156.

Hounsgaard J, Hultborn H, Jespersen B & Kiehn O (1988). Bistability of alpha-motoneurones in the decerebrate cat and in the acute spinal cat after intravenous 5-hydroxytryptaphan. J Physiol 405, 345–367.

Hudson AL, Butler JE, Gandevia SC & De Troyer A (2010). Interplay between the inspiratory and postural functions of the human parasternal intercostal muscles. J Neurophysiol 103, 1622–1629.

Hudson AL, Butler JE, Gandevia SC & de Troyer A (2011a). Role of the diaphragm in trunk rotation in humans. J Neurophysiol 106, 1622–1628.

Hudson AL, Gandevia SC & Butler JE (2011b). Common rostrocaudal gradient of output from human intercostal motoneurones during voluntary and automatic breathing. Respir Physiol Neurobiol 175, 20–28.

Hudson AL, Gandevia SC & Butler JE (2017). Task-dependent output of human parasternal intercostal motor units across spinal levels. J Physiol 595, 7081–7092.

Hultborn H, Denton ME, Wienecke J & Nielsen JB (2003). Variable amplification of synaptic input to cat spinal motoneurones by dendritic persistent inward current. J Physiol 552, 945–952.

Jenz ST, Beauchamp JA, Gomes MM, Negro F, Heckman CJ & Pearcey GEP (2023). Estimates of persistent inward currents in lower limb motoneurons are larger in females than in males. J Neurophysiol 129, 1322–1333.

Lapole T, Mesquita RNO, Baudry S, Souron R, O'Brien EK, Brownstein CG & Rozand V (2024). Persistent inward currents in tibialis anterior motoneurons can be reliably estimated within the same session. Journal of Electromyography and Kinesiology 78, 102911.

Lee RH & Heckman CJ (1999). Paradoxical effect of QX-314 on persistent inward currents and bistable behavior in spinal motoneurons in vivo. J Neurophysiol 82, 2518–2527.

Lecce, E., del Vecchio, A., Nuccio, S., Felici, F., & Bazzucchi, I. (2025). Higher dominant muscle strength is mediated by motor unit discharge rates and proportion of common synaptic inputs. Scientific Reports 2025 15:1, 15(1), 8269.

Li Y, Gorassini MA & Bennett DJ (2004). Role of Persistent Sodium and Calcium Currents in Motoneuron Firing and Spasticity in Chronic Spinal Rats. J Neurophysiol 91, 767–783.

Mackay K, Orssatto LBR, Polman R, Van der Pols JC & Trajano GS (2023). Caffeine does not influence persistent inward current contribution to motoneuron firing. J Neurophysiol 130, 1529-1540.

Manuel M, Li Y, ElBasiouny SM, Murray K, Griener A, Heckman CJ & Bennett DJ (2012). NMDA induces persistent inward and outward currents that cause rhythmic bursting in adult rodent motoneurons. J Neurophysiol 108, 2991–2998.

Mesquita RNO, Taylor JL, Heckman CJ, Trajano GS & Blazevich AJ (2024). Persistent inward currents in human motoneurons: Emerging evidence and future directions. J Neurophysiol 132, 1278–1301.

Mesquita RNO, Taylor JL, Trajano GS, Škarabot J, Holobar A, Gonçalves BAM & Blazevich AJ (2022). Effects of reciprocal inhibition and whole-body relaxation on persistent inward currents estimated by two different methods. Journal of Physiology 600, 2765–2787.

Mesquita RNO, Taylor JL, Trajano GS, Holobar A, Gonçalves BAM & Blazevich AJ (2023). Effects of jaw clenching and mental stress on persistent inward currents estimated by two different methods. European Journal of Neuroscience 58, 4011–4033.

Mohammadalinejad G, Afsharipour B, Yacyshyn A, Duchcherer J, Bashuk J, Bennett E, Pearcey GEP, Negro F, Quinlan KA, Bennett DJ & Gorassini MA (2024). Intrinsic motoneuron properties in typical human development. J Physiol 602, 2061-2087.

Orssatto LBR, Fernandes GL, Blazevich AJ & Trajano GS (2022). Facilitation–inhibition control of motor neuronal persistent inward currents in young and older adults. Journal of Physiology 600, 5101–5117.

Orssatto LBR, Borg DN, Blazevich AJ, Sakugawa RL, Shield AJ & Trajano GS (2021a). Intrinsic motoneuron excitability is reduced in soleus and tibialis anterior of older adults. Geroscience 43, 2719–2735.

Orssatto LBR, Mackay K, Shield AJ, Sakugawa RL, Blazevich AJ & Trajano GS (2021b). Estimates of persistent inward currents increase with the level of voluntary drive in low-threshold motor units of plantar flexor muscles. J Neurophysiol 125, 1746–1754.

Pearcey GEP, Khurram OU, Beauchamp JA, Negro F & Heckman CJ (2022). Antagonist tendon vibration dampens estimates of persistent inward currents in motor units of the human lower limb. bioRxiv2022.08.02.502526.

Powers RK & Heckman CJ (2017). Synaptic control of the shape of the motoneuron pool input-output function. J Neurophysiol 117, 1171–1184.

Revill AL & Fuglevand AJ (2011). Effects of persistent inward currents, accommodation, and adaptation on motor unit behavior: A simulation study. J Neurophysiol 106, 1467–1479.

Seven YB, Mantilla CB, Zhan WZ & Sieck GC (2013). Non-stationarity and power spectral shifts in EMG activity reflect motor unit recruitment in rat diaphragm muscle. Respir Physiol Neurobiol 185, 400–409.

Shapiro NP & Lee RH (2007). Synaptic amplification versus bistability in motoneuron dendritic processing: A top-down modeling approach. J Neurophysiol 97, 3948–3960.

Sieck GC & Fournier M (1989). Diaphragm motor unit recruitment during ventilatory and nonventilatory behaviors. J Appl Physiol 66, 2539–2545.

Svirskis G & Hounsgaard J (1997). Depolarization-Induced Facilitation of a Plateau-Generating Current in Ventral Horn Neurons in the Turtle Spinal Cord. J Neurophysiol 78, 1740–1742.

Udina E, D'Amico J, Bergquist AJ & Gorassini MA (2010). Amphetamine Increases Persistent Inward Currents in Human Motoneurons Estimated From Paired Motor-Unit Activity. J Neurophysiol 103, 1295–1303.

Yacyshyn, A. F., Mohammadalinejad, G., Afsharipour, B., Duchcherer, J., Bashuk, J., Bennett, D. J., Negro, F., Quinlan, K. A., & Gorassini, M. A. (2025). Sex-related differences in motoneuron firing behavior during typical development. Journal of Neurophysiology, 133(4), 1307–1319.

Zero AM, Kirk EA & Rice CL (2022). Firing rate trajectories of human motor units during activity-dependent muscle potentiation. J Appl Physiol 132, 402–412.

END OF COMMENTS

Dear Dr Mesquita,

Re: JP-RP-2026-290663R1 "**Estimation of persistent inward currents contribution to inspiratory motoneuron firing in humans**" by Ricardo N. O. Mesquita, Simon C Gandevia, Janet L Taylor, Anna L Hudson, and Jane Butler

We are pleased to tell you that your paper has been accepted for publication in The Journal of Physiology.
- Please ensure that any tables are editable and in Word format and embedded in the article file itself. Currently, it is uploaded as "Supporting Information for online publication (Data set/Video/Audio only)". Please add editable tables to the Word file of your manuscript and remove "Supporting Information for online publication (Data set/Video/Audio only)" files.

- Please include an Abstract Figure legend. An appropriate figure legend, which should not exceed 150 words in length, should be included in the main manuscript file.

The last Word (or similar) version of the manuscript provided will be used by the Production Editor to prepare your proof. When this is ready you will receive an email containing a link to Wiley's Online Proofing System. The proof should be thoroughly checked and corrected as promptly as possible.

Authors should note that it is too late at this point to offer corrections prior to proofreading. Major corrections at proof stage, such as changes to figures, will be referred to the Editors for approval before they can be incorporated. Only minor changes, such as to style and consistency, should be made at proof stage. Changes that need to be made after proof stage will usually require a formal correction notice.

All queries at proof stage should be sent to: TJP@wiley.com.

Following copyediting and typesetting the accepted version will be published online.

Yours sincerely,

Richard Carson
Senior Editor
The Journal of Physiology

IMPORTANT POINTS TO NOTE FOLLOWING ACCEPTANCE OF YOUR PAPER:

• IMPORTANT NOTICE ABOUT OPEN ACCESS: To assist authors whose funding agencies mandate immediate public access to published research findings, The Journal of Physiology allows authors to pay an Open Access (OA) fee to have their papers made freely available immediately on publication.

The Corresponding Author will receive an email from Wiley with details on how to register or log in to Wiley Authors where you will be able to place an order.

• You can check if your funder or institution has a Wiley Open Access Account here:
https://authors.wiley.com/author-resources/Journal-Authors/open-access/author-compliance-tool.html

• TRANSPARENT PEER REVIEW POLICY: To improve the transparency of its peer review process, The Journal of Physiology publishes online as supporting information the peer review history of all articles accepted for publication. Readers will have access to decision letters, including Editors' comments and referee reports, for each version of the manuscript as well as any author responses to peer review comments. Referees can decide whether or not they wish to be named on the peer review history document.

• You can help your research get the attention it deserves! Check out Wiley's free Promotion Guide for best-practice recommendations for promoting your work at: www.wileyauthors.com/eeo/guide. You can learn more about Wiley Editing Services which offers professional video, design, and writing services to create shareable video abstracts, infographics, conference posters, lay summaries, and research news stories for your research at: www.wileyauthors.com/eeo/promotion.

• If you would like to receive our 'Research Roundup', a monthly newsletter highlighting the cutting-edge research published in The Physiological Society's family of journals (The Journal of Physiology, Experimental Physiology, Physiological Reports, The Journal of Nutritional Physiology and The Journal of Precision Medicine: Health and Disease), please click this link, fill in your name and email address and select 'Research Roundup':
https://www.physoc.org/journals-and-media/membernews

REFEREE COMMENTS

Referee #1:

Thank you for being very responsive to my previous comments and recommendations.

Referee #2:

The authors have addressed all concerns. Modifications have greatly increased the clarity and quality of the manuscript. There are no further concerns.
